# β‐Glucuronidase‐Expressing *Lactobacillus reuteri* Triggers Irinotecan Enterotoxicity Through Depleting the Regenerative Epithelial Stem/Progenitor Pool

**DOI:** 10.1002/advs.202411052

**Published:** 2025-04-26

**Authors:** Bei Yue, Ruiyang Gao, Ling Zhao, Donghui Liu, Cheng Lv, Ziyi Wang, Fangbin Ai, Beibei Zhang, Zhilun Yu, Xiaolong Geng, Hao Wang, Kang Wang, Kaixian Chen, Chenghai Liu, Zhengtao Wang, Wei Dou

**Affiliations:** ^1^ The MOE key Laboratory of Standardization of Chinese Medicines Shanghai Key Laboratory of Compound Chinese Medicines and the SATCM key Laboratory for New Resources and Quality Evaluation of Chinese Medicines Institute of Chinese Materia Medica Shanghai University of Traditional Chinese Medicine Shanghai 201203 China; ^2^ School of Integrative Medicine Shanghai University of Traditional Chinese Medicine Shanghai 201203 China; ^3^ Centre for Chinese Herbal Medicine Drug Development Limited Hong Kong Baptist University Hong Kong SAR China; ^4^ Department of Hepatology Shuguang Hospital Affiliated to Shanghai University of Traditional Chinese Medicine Shanghai 201203 China

**Keywords:** epithelial regeneration, irinotecan‐induced intestinal toxicity, GUS‐expressing bacteria, lactobacillus reuteri, Xianglian pill

## Abstract

Irinotecan (CPT11)‐induced diarrhea affects 80–90% of cancer patients due to β‐glucuronidase (GUS) converting 7‐ethyl‐10‐hydroxycamptothecin glucuronide (SN38G) to 7‐ethyl‐10‐hydroxycamptothecin (SN38). It remains unclear whether SN38 impacts the homeostasis between gut microbiota and mucosal stem cell niche. This study explores the crosstalk between gut microbiota and intestinal stem cells (ISCs) in intestinal mucositis triggered by CPT11 chemotherapy. CPT11‐treated mice exhibited significant colon shortening, inflammatory infiltration, intestinal barrier dysfunction, and ISC impairment, which correlated with gut dysbiosis, enrichment of GUS‐expressing bacteria, and intraluminal SN38 accumulation. In contrast, antidiarrheal (Xianglian pill) treatment alleviated SN38‐induced enterotoxicity and reduced GUS‐expressing bacterial populations. Microbiome profiling of clinical patients and mucositis mice revealed a strong correlation between CPT11/SN38 enterotoxicity and GUS‐expressing bacteria, particularly *Lactobacillus reuteri*. PLS‐PM modeling further linked *L. reuteri* to impaired epithelial regeneration, which is validated using a 3D intestinal organoid model. *L. reuteri* hindered ISC differentiation into secretory lineages within the organoids. Furthermore, *L. reuteri* colonization in mice exacerbated mucositis and disrupted epithelial differentiation, while its elimination ameliorated colitis symptoms and preserved crypt cell stemness. These findings suggest that selectively targeting GUS‐expressing bacteria, particularly L. reuteri, to protect the regenerative epithelial stem/progenitor pool may serve as an effective strategy for mitigating CPT11‐induced enterotoxicity.

## Graphical abstract

XLP mitigated CPT11 mucositis by suppressing GUS‐expressing microbes, notably *L. reuteri*, and diminishing bacterial GUS activity, consequently reducing SN38 accumulation to protect the intestinal epithelium. This preservation of the mucosal stem cell niche enabled rapid regeneration of secretory lineages such as mucin‐producing goblet cells, which fortify the epithelial barrier.

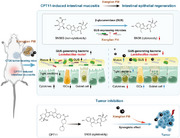

## 1 Introduction

Irinotecan (CPT11), a semisynthetic derivative of the natural alkaloid camptothecin, is widely used in the treatment of advanced colorectal cancer and other solid tumors.^[^ ^1^ ^]^ However, its clinical utility is severely limited by severe gastrointestinal (GI) toxicity, which imposes a significant burden on cancer patients.^[^ ^2^ ^]^ Following intravenous administration, CPT11 is metabolized in the GI tract into its active metabolite, 7‐ethyl‐10‐hydroxycamptothecin (SN38), which is highly cytotoxic to intestinal epithelial cells, leading to severe complications such as diarrhea and mucositis.^[^ ^3^ ^]^ Notably, 50–80% of patients receiving standard CPT11 chemotherapy experience GI toxicity, with ≈20% developing severe mucositis, which can progress to life‐threatening conditions such as bacteremia and dehydration.^[^ ^4^ ^]^ Current clinical management strategies include antidiarrheal agents, anticholinergic drugs, and antibiotics.^[^ ^5^ ^]^ However, these treatments primarily provide temporary symptomatic relief and may lead to further complications, including intestinal obstruction and antibiotic resistance. These limitations highlight the urgent need for more effective and sustainable therapeutic strategies to mitigate CPT11‐induced GI side effects.

The intestinal epithelium, serving as a crucial barrier between the host and the external environment, comprises enterocytes, goblet cells, and stem cells, all of which play essential roles in maintaining gut homeostasis.^[^ ^6^ ^]^ During CPT11 chemotherapy, patients frequently experience severe diarrhea and epithelial damage.^[^ ^7^ ^]^ CPT11 disrupts epithelial integrity and impairs regenerative capacity, particularly affecting goblet cells and intestinal stem cells (ISCs).^[^ ^8^ ^]^ Goblet cells, which are responsible for mucus production, and ISCs, which drive epithelial renewal, are particularly vulnerable to CPT11‐induced cytotoxicity.^[^ ^9^ ^]^ Their depletion compromises mucosal barrier function and reduces intestinal regenerative potential, significantly exacerbating CPT11‐induced GI toxicity. This underscores the need for further research into effective therapeutic strategies to mitigate chemotherapy‐induced epithelial injury and preserve intestinal integrity. A critical factor contributing to the adverse effects of CPT11 is the gut microbiota.^[^ ^10^ ^]^ Emerging evidence indicates that gut microbiota modulates intestinal toxicity during chemotherapy.^[^ ^11^ ^]^ CPT11, a prodrug, is initially metabolized by carboxylesterases (CES) into SN38, a metabolite that is 100 to 1000 times more cytotoxic than CPT11 itself.^[^ ^12^ ^]^ SN38 is then converted into its inactive glucuronidated form (SN38G) by uridine diphosphate‐glucuronosyltransferase (UGT) in the liver and intestine, before being excreted into the GI tract. However, certain gut bacteria produce β‐glucuronidase (GUS), which hydrolyzes SN38G back into active SN38, leading to its local accumulation in the gut lumen. This excessive SN38 concentration worsens epithelial damage and inflammatory responses, ultimately resulting in severe diarrhea and mucositis.^[^ ^13^ ^]^ Additionally, CPT11 treatment disrupts gut microbiota balance, promoting an overgrowth of pro‐inflammatory and GUS‐expressing bacteria, while depleting beneficial commensal microbes.^[^ ^14^ ^]^ Thus, a deeper understanding of the complex interplay between the gut microbiota and intestinal mucosal epithelium is essential for developing microbiota‐targeted therapies to alleviate CPT11‐induced GI toxicity and enhance treatment outcomes in cancer patients.

 Recognizing the crucial role of bacterial‐derived GUS in SN38 regeneration, the development of GUS inhibitors has emerged as a potential strategy to alleviate CPT11 chemotoxicity.^[^ ^15^ ^]^ Various GUS inhibitors, including amoxapine, antibiotics (penicillin, neomycin, streptomycin, ciprofloxacin), herbal compounds (baicalin, luteolin, paeoniflorin), and synthesized molecules, have demonstrated protective effects against CPT11‐induced GI adverse reactions.^[^ ^16^, ^17^, ^18^, ^19^ ^]^ However, current therapeutic strategies primarily focus on reducing inflammation and inhibiting GUS enzyme activity, while overlooking the role of GUS‐expressing bacteria, the regeneration of intestinal epithelium, and the complex interactions between them. Notably, up to 43% of bacterial species in the gut produce GUS, predominantly from the genera *Bacteroidetes*, *Firmicutes*, and *Proteobacteria*.^[^ ^17^ ^]^ While broad‐spectrum antibiotics have been used to deplete gut bacteria, this approach has shown limited clinical efficacy and is associated with increased infection risk, antibiotic resistance, and gut microbiota dysbiosis.^[^ ^20^ ^]^ Thus, there is a pressing need for a conceptual shift from a “one‐size‐fits‐all” antibacterial strategy to the selective suppression of GUS‐expressing bacteria, which may provide a more effective and targeted intervention for chemotherapy‐associated GI toxicity. However, few studies have specifically investigated the influence of GUS‐expressing bacteria on chemotoxicity.

Xianglian pill (XLP), a well‐known traditional medicine composed of *Rhizoma Coptidis* and *Aucklandiae Radix*, has long been used for the treatment of abdominal pain and diarrhea.^[^ ^21^ ^]^ Notably, both *R. Coptidis* and *A. Radix* are recognized in the European Pharmacopoeia.^[^ ^22^ ^]^ Recent studies have demonstrated the efficacy of XLP in alleviating antibiotic‐induced diarrhea and reducing dextran sulfate sodium (DSS)‐induced intestinal injury, suggesting its potential as an adjuvant therapy for CPT11‐induced enterotoxicity.^[^ ^23^, ^24^ ^]^ Growing evidence suggests that targeted modulation of gut microbiota can significantly impact GI adverse reactions caused by chemotherapeutic drugs.^[^ ^17^ ^]^ However, the specific role of GUS‐expressing bacteria in mediating CPT11‐associated GI toxicity remains to be elucidated. In this study, we utilized XLP as a potential antidiarrheal drug to investigate the role of GUS‐generating bacteria in the treatment of CPT11‐induced intestinal mucositis, as well as the crosstalk between GUS‐expressing bacteria and ISCs in the progression of mucositis. We hypothesize that CPT11‐induced enterotoxicity is driven by the overgrowth of GUS‐expressing bacteria and the resultant upregulation of bacterial GUS activity, leading to excessive production and accumulation of SN38 in the intestinal tract, thereby impairing the epithelial regenerative system. Our findings uncover a novel mechanism by which XLP mitigates CPT11‐induced enterotoxicity through modulation of the “GUS microbe‐host‐irinotecan” axis. From a fundamental research perspective, our findings underscore the crucial role of GUS‐expressing bacteria, particularly *Lactobacillus reuteri*, in mediating CPT11‐induced enterotoxicity and depleting the intestinal stem/progenitor cell pool. Furthermore, selectively targeting GUS‐expressing bacteria to enhance ISC proliferation, renewal, and differentiation may offer an effective therapeutic strategy for managing chemotherapy‐induced GI adverse effects.

## 2 Results

### 2.1 XLP, as a Potential Antidiarrheal Drug, Alleviates CPT11‐Induced Intestinal Mucositis and Reinforces Intestinal Barrier Integrity

**Figure 1:**
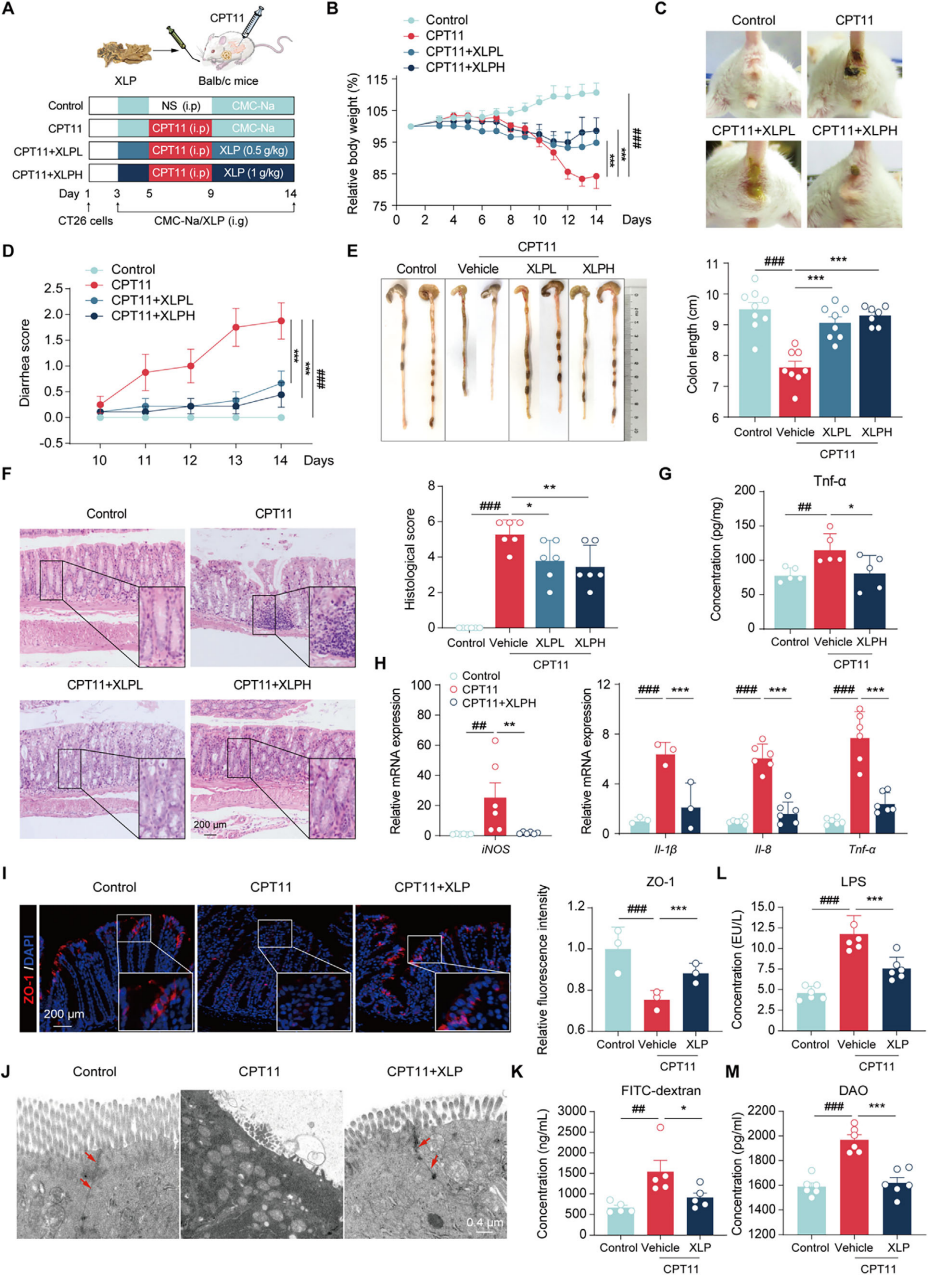
XLP alleviated CPT11‐induced intestinal toxicity in colorectal tumor xenograft mice. A) Experimental design. B) Body weight changes (*n* = 6–8). C) Representative anus photographs. D) Percentage of mice with bloody diarrhea over time (*n* = 6). E) Representative colon photographs and length measurements (*n* = 6). F) Colonic sections by H&E staining and histological scoring (*n* = 6). G) Serum Tnf‐α concentrations (*n* = 5). H) Relative mRNA levels of iNOS, IL‐1β, IL‐8, and Tnf‐α in colon tissues (*n* = 3–6). I) ZO‐1 immunofluorescence images and quantification in colon tissues (scale bar = 200 µm). J) Ultrastructural images of intestinal villi and tight junctions (red arrows) via transmission electron microscopy (scale bars = 0.4 µm). K) Serum concentrations of FITC‐dextran (*n* = 5). L, M) Serum LPS and DAO levels measured by ELISA. Data are expressed as mean ± SEM (B) or mean ± SD (D‐I, K‐M). Statistical analysis was performed using one‐way ANOVA (B, D‐I, K‐M). ^##^ *p* < 0.01, ^###^ *p* < 0.001 versus control group; *p < 0.05, **p < 0.01, ***p < 0.001 versus CPT11 group.

The potential anticolitis activity of the antidiarrheal agent XLP was assessed in colorectal tumor‐bearing mice with CPT11‐induced intestinal mucositis (**Figure** **1**A). Mice receiving intraperitoneal CPT11 injections (60 mg kg^−1^ day^−1^) for five consecutive days exhibited significant weight loss and persistent diarrhea (Figure 1B–D). Oral administration of XLP at 0.5 and 1.0 g kg^−1^ notably alleviated weight loss and diarrhea. Colon shortening, a key indicator of mucosal inflammation, was significantly induced by CPT11 but mitigated by XLP treatment (Figure 1E). Macroscopic and histological analyses revealed severe pathological lesions in CPT11‐treated mice, including mucosal erosion, epithelial disruption, and neutrophil infiltration (Figure 1F). However, XLP treatment significantly preserved mucosal architecture and reduced inflammatory cell infiltration in the affected mice. Notably, the 1.0 g kg^−1^ dosage group demonstrated greater efficacy than the 0.5 g kg^−1^ group, leading to the selection of XLPH (1.0 g kg^−1^) for further experimental studies.

Additionally, XLP reduced the CPT11‐induced elevation of serum tumor necrosis factor (Tnf)‐α and suppressed the expression of pro‐inflammatory markers, including inducible nitric oxide synthase (iNOS), interleukin (IL)‐1β, IL‐8, Tnf‐α, and cyclooxygenase (Cox)‐2 in colon tissue (Figure 1G,H; Figure S1A, Supporting Information). XLP also preserved intestinal epithelial barrier integrity by restoring mRNA and protein levels of tight junction proteins, such as zonula occludens‐1 (ZO‐1), Occludin, and Claudin‐7 in colon tissue (Figure S1B, C, Supporting Information). The protective effects of XLP on brush border structure, intestinal villi, and tight junctions were further confirmed by immunofluorescence staining for ZO‐1 and transmission electron microscopy (Figure 1I,J). Functional assays demonstrated that XLP mitigated CPT11‐induced intestinal permeability, as indicated by reduced FITC‐dextran translocation and lower serum levels of lipopolysaccharide (LPS) and diamine oxidase (DAO) (Figure 1K–M).

In vitro, the effects of XLP on the viability of NCM460 human colonic epithelial cells were assessed using the cell counting kit‐8 (CCK‐8) assay. XLP exhibited no cytotoxicity within the tested concentration range of 0–100 µg mL^−1^ (Figure S1D, Supporting Information). The scratch wound‐healing assay demonstrated that XLP facilitated the repair of cell wounds induced by the active CPT11 metabolite SN38 (Figure S1E, Supporting Information). Furthermore, the transepithelial electric resistance (TEER) assay revealed that XLP significantly mitigated the SN38‐induced reduction in intestinal epithelial cell resistance after 12 or 24 h of exposure (Figure S1F, Supporting Information). Additionally, XLP effectively counteracted the SN38‐induced reduction in the expression of tight junction proteins ZO‐1, Occludin, and Claudin‐1 (Figure S1G, Supporting Information).

Collectively, these findings indicate that CPT11 induces significant intestinal inflammation and epithelial damage. However, XLP effectively mitigates CPT11‐induced inflammatory responses and intestinal barrier disruption, likely by strengthening barrier integrity and promoting wound healing.

### 2.2 CPT11‐Induced Enterotoxicity is Characterized by Disrupted Intestinal Epithelial Homeostasis, Which is Effectively Restored by XLP

**Figure 2:**
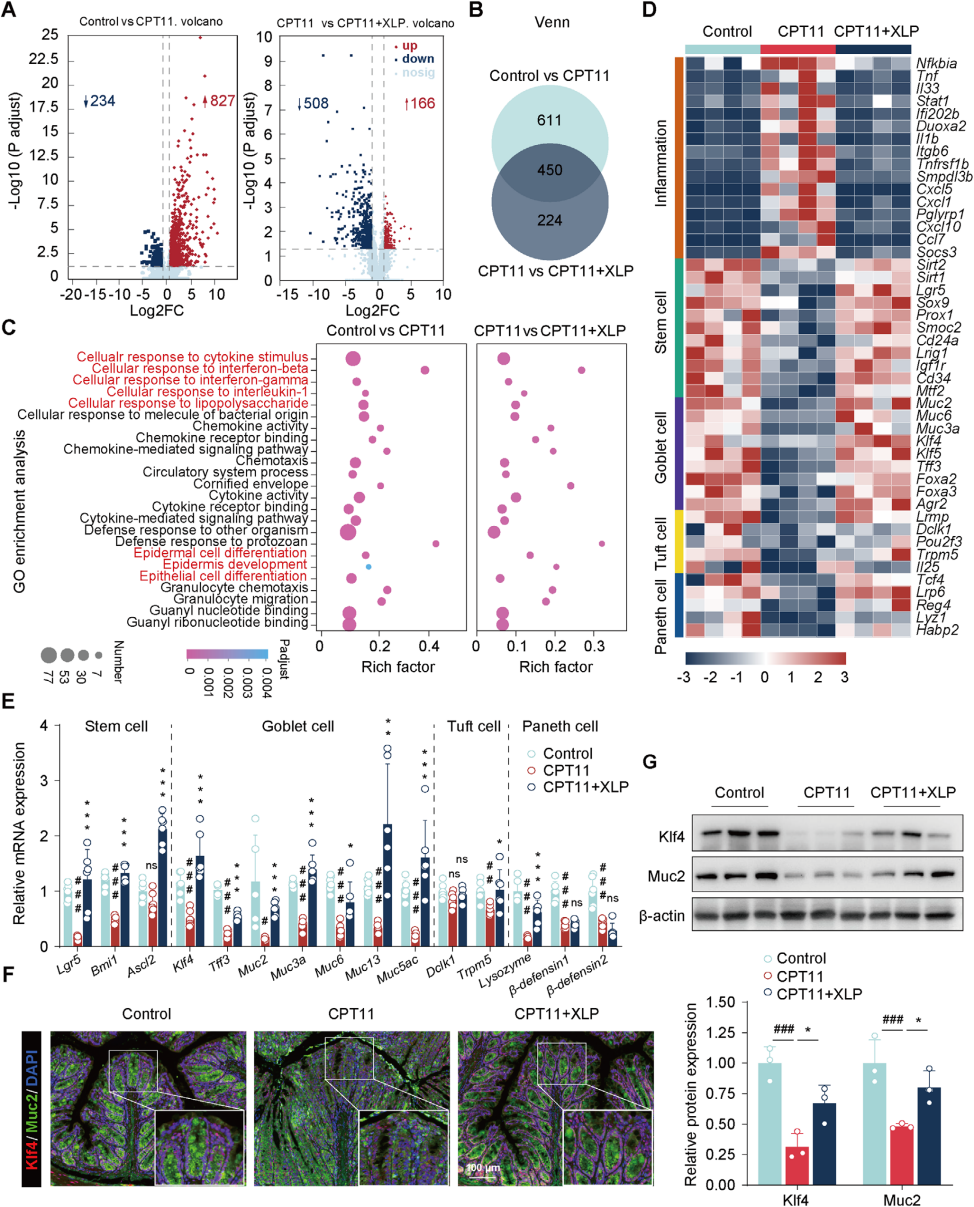
XLP promoted intestinal epithelial differentiation in CPT11 mucositis mice. A) Volcano plot of differentially expressed genes in the colon by DESeq2 analysis. Blue dots: down‐regulated genes (fold change < −1), red dots: up‐regulated genes (fold change > 1), laurel‐green dots: no significant difference (*n* = 4). B) Venn diagram of colonic genes significantly recovered by XLP treatment (*n* = 4). C) Gene Ontology (GO) enrichment analysis of differentially expressed genes. Color intensity: adjusted p‐value, node size: number of genes in category (*n* = 4). D) Heatmap of marker gene expression for various cell types. (*n* = 4). E) Relative mRNA levels of markers for different cell types in colon tissues (*n* = 6). F) Immunofluorescence images of Klf4 (red) and Muc2 (green) in colon tissues (*n* = 3, scale bar: 100 µm). Nuclei labeled with DAPI (blue). G) Representative western blots and quantitative analysis of Klf4 and Muc2 in colon tissues (*n* = 3). Data are expressed as mean ± SD. Statistical analysis was performed using one‐way ANOVA (E, G). ^#^ *p* < 0.05, ^##^ *p* < 0.01, ^###^ *p* < 0.001 versus control group; *p < 0.05, **p < 0.01, ***p < 0.001 versus CPT11 group; ns, no significance.

Intestinal epithelial homeostasis relies on a delicate balance among the intestinal microecology, mucosal barrier, and cellular components. Central to this balance is the self‐renewal and differentiation of ISCs, which are crucial for maintaining equilibrium and driving the continuous renewal of the intestinal epithelium and its specialized cell types.^[^ ^6^ ^]^ To explore the molecular mechanisms underlying CPT11‐induced disruption of intestinal epithelial homeostasis, we performed comprehensive transcriptomic analysis of whole colon tissue using RNA sequencing. CPT11 treatment led to significant gene expression alterations, with 234 genes downregulated and 827 genes upregulated compared to control mice (fold change > 2, P < .05) (**Figure** **2**A). In contrast, XLP treatment resulted in the upregulation of 166 genes and downregulation of 508 genes compared to CPT11 treatment alone. Notably, XLP restored the expression of 450 out of the 1061 genes significantly altered by CPT11, suggesting its role in reversing CPT11‐induced transcriptional dysregulation (Figure 2B).

Functional enrichment analysis using the Gene Ontology (GO) database highlighted the impact of XLP treatment on inflammation‐related processes, including cytokine response pathways and epithelial cell differentiation (Figure 2C). Heatmap analysis further revealed a significant upregulation of inflammation‐related genes in CPT11‐treated mice, which was partially reversed by XLP treatment (Figure 2D). Additionally, CPT11 exposure led to a marked reduction in the expression of genes associated with ISCs and secretory‐lineage cells, including goblet cells, tuft cells, and Paneth cells (Figure 2D). However, XLP effectively restored the expression levels of these genes. RT‐qPCR analysis confirmed that XLP treatment significantly increased the mRNA expression of markers related to stem cells, goblet cells, tuft cells, and Paneth cells in CPT11‐treated mice, though the differences in tuft cell and Paneth cell markers were not statistically significant (Figure 2E).

**Figure 3:**
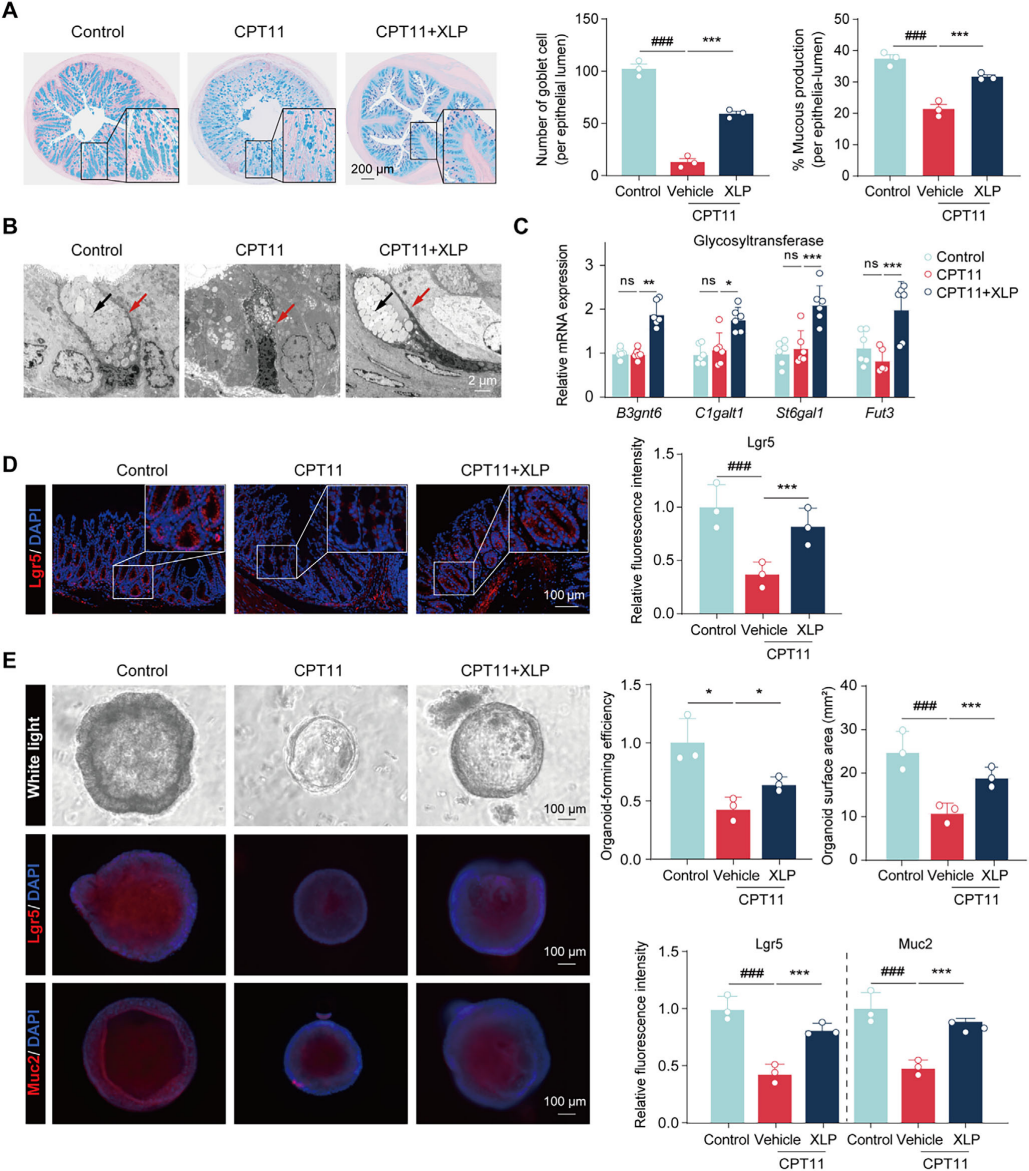
XLP enhanced goblet cell function and ISC differentiation into goblet cells in CPT11 mucositis mice. A) AB‐PAS staining of colonic sections (scale bar: 200 µm) and quantitative analysis of goblet cells and mucus production (*n* = 3). B) Transmission electron microscopy of showing mucin particles (black arrows) and goblet cells (red arrows, scale bar: 2 µm). C) Relative mRNA levels of glycosyltransferase genes in colon tissues (*n* = 6). D) Immunofluorescence images and quantification of Lgr5 (red) in colon tissues (*n* = 3, scale bar: 100 µm). Nuclei labeled with DAPI (blue). E) Bright‐field and immunofluorescence images of Muc2 and Lgr5 in colonic organoids. Qualitative statistics of organoid‐forming efficiency, surface area, and fluorescence intensity of Lgr5 and Muc2 (scale bar: 100 µm) (*n* = 3). Data are expressed as Mean ± SD. Statistical analysis was performed using one‐way ANOVA (A, C‐E). ^###^ *p* < 0.001 versus control group; *p < 0.05, ***p < 0.001 versus CPT11 group; ns, no significance.

Goblet cells play a crucial role in mucus production and maintaining intestinal mucosal integrity.^[^ ^25^ ^]^ Immunofluorescence staining and western blot analysis revealed a reduction in key goblet cell markers, including Krüppel‐like factor 4 (Klf4) and mucin 2 (Muc2), in the colonic crypts of CPT11‐treated mice, which was partially restored by XLP treatment (Figure 2F,G). Assessment of mucus secretion and goblet cell abundance showed a significant decrease in mucus‐secreting goblet cells following CPT11 exposure, which was markedly reversed by XLP treatment (**Figure** **3**A). Scanning electron microscopy further confirmed the absence of mucus particles in goblet cells of CPT11‐treated mice, whereas XLP‐treated mice exhibited normal mucus production (Figure 3B). RT‐qPCR analysis demonstrated increased mRNA expression of key glycosyltransferase genes involved in mucin glycoprotein synthesis, including B3gnt6 (UDP‐GlcNAc β‐1,3‐N‐acetylglucosaminyltransferase 6), C1galt1 (core 1 synthase glycoprotein‐N‐acetylgalactosamine 3‐β‐galactosyltransferase 1), St6gal1 (ST6 β‐galactoside α‐2,6‐sialyltransferase), and Fut3 (fucosyltransferase 3), in XLP‐treated mice compared to CPT11‐exposed mice (Figure 3C). These findings suggest that XLP may enhance goblet cell function by promoting glycosyltransferase expression and mucus secretion.

We hypothesize that the beneficial effects of XLP on goblet cell secretion may result from its protective role in intestinal crypt stem cells. To investigate this, immunofluorescence staining was performed to assess leucine‐rich repeat‐containing G‐protein‐coupled receptor 5 (Lgr5)‐positive crypt cells. CPT11 exposure significantly diminished Lgr5 fluorescence intensity in colonic crypts, whereas XLP treatment partially restored Lgr5 expression (Figure 3D), indicating that XLP alleviates ISC impairment. To further evaluate the impact of CPT11 and CPT11 combined with XLP on ISC proliferation, we established an in vitro 3D organoid model using isolated colonic crypt cells. Organoid‐forming efficiency and surface area were significantly reduced in the CPT11 group compared to healthy controls (Figure 3E). However, XLP treatment enhanced both parameters in CPT11‐exposed mice. Additionally, XLP mitigated CPT11‐induced organoid damage, restoring the expression of Lgr5 and Muc2 (Figure 3E, bottom right panel), further supporting its role in promoting ISC proliferation and differentiation.

Collectively, these findings indicate that CPT11‐induced enterotoxicity disrupts epithelial homeostasis, characterized by exacerbated colonic inflammation and impaired ISC differentiation. However, XLP treatment effectively suppresses colonic inflammation while enhancing ISC self‐renewal and differentiation, thereby promoting intestinal epithelial repair.

### 2.3 CPT11 Triggers Gut Microbiota Dysbiosis by Increasing the Population of GUS‐Expressing Bacteria, Which is Reversed by XLP Treatment

**Figure 4:**
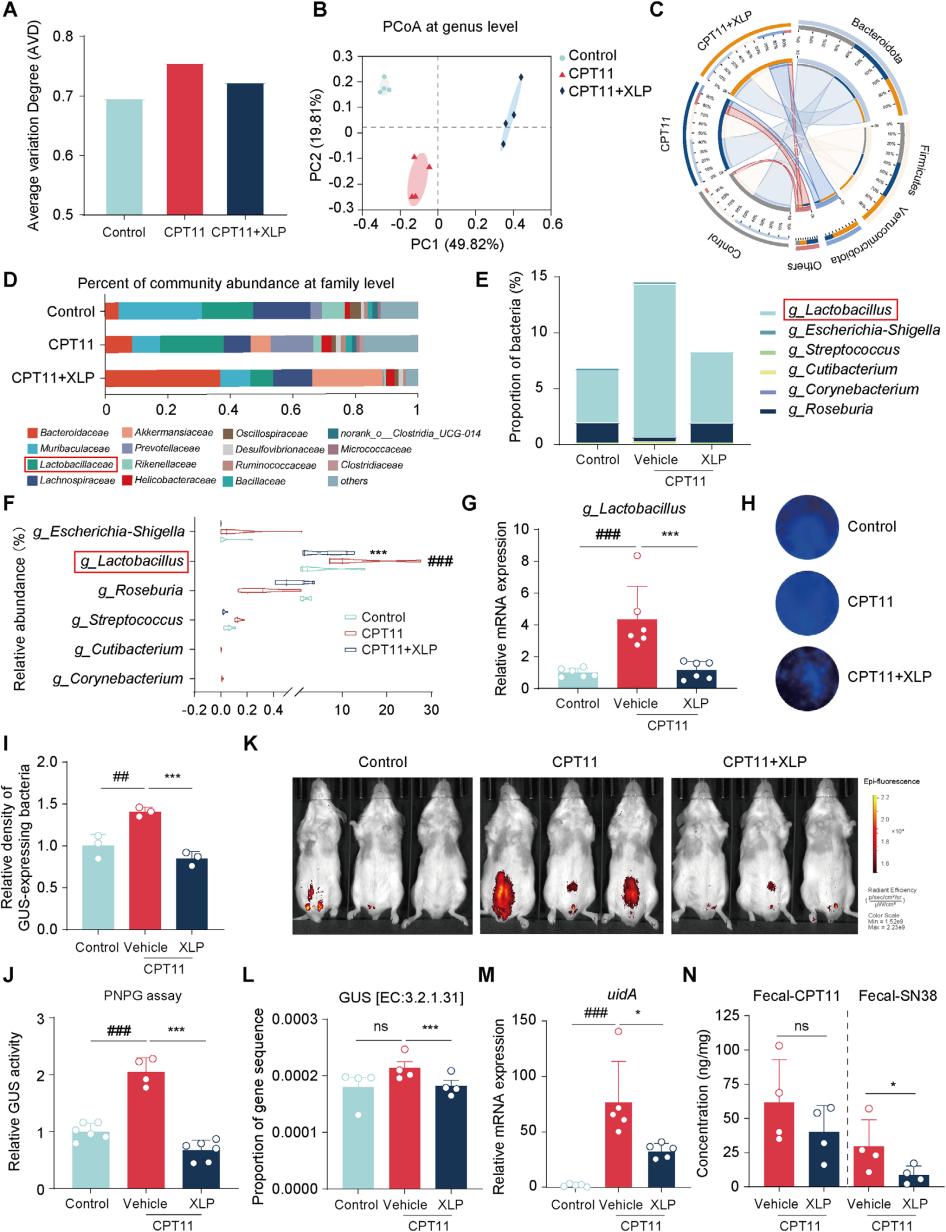
XLP suppressed GUS‐producing bacteria and reduced GUS activity in CPT11 mucositis mice. A) The Average Variation Degree (AVD) index analysis. B) Principal coordinates analysis (PCoA) of different group samples at the OTU level (*n* = 4). C) Circos analysis showing abundance relationships between groups and bacterial communities at the phylum level. D) Percent community abundance of differentially expressed bacteria at the family level (*n* = 4). E) Stacked bar chart of GUS‐producing bacteria at the genus level (*n* = 4). F) Violin plots of GUS‐producing bacteria distribution at the genus level. Lines within each violin represents the median (*n* = 4). G) mRNA level of *g_Lactobacillus* in feces (*n* = 6). H, I) Images of GUS‐producing bacteria in feces using 4‐MUG supplemented agar, with blue indicating abundance. J) GUS activity in feces detected by PNPG assay (*n* = 4). K) Intestinal GUS activity visualized by in vivo imaging, with red to yellow indicating high to low activity (*n* = 3). L) Proportional abundance of ortholog K01195 (GUS) according to KEGG database (*n* = 4). M) mRNA levels of uidA gene in feces (*n* = 5). N) Concentrations of CPT11 and SN38 in feces 24 h after the last XLP dose (*n* = 4). Data are expressed as Mean ± SD. Statistical analysis was performed using one‐way ANOVA (F‐G, I‐J, L‐M) or an unpaired Student's *t*‐test (N). ^##^ *p* < 0.01, ^###^ *p* < 0.001 versus control group; *p < 0.05, ***p < 0.001 versus CPT11 group; ns, no significance.

The gut microbiota plays an indispensable role in in various physiological processes, including facilitating digestion, supporting immune system development, and protecting against pathogenic organisms.^[^ ^26^ ^]^ Maintaining a diverse and well‐balanced microbial ecosystem is essential for immune regulation and disease prevention.^[^ ^27^ ^]^ To evaluate the impact of CPT11 on the gut microbiota, we performed high‐throughput sequencing of the V3–V4 hypervariable regions of the 16S rRNA gene in fecal samples. The Average Variation Degree (AVD) index showed a significant increase in the CPT11 group, indicating reduced microbial community stability post‐treatment (**Figure** **4**A). In contrast, XLP administration improved microbial stability, as evidenced by a lower AVD index. Principal Coordinates Analysis (PCoA) revealed distinct microbial compositions between the CPT11 and normal control groups, while the microbiota profile of the CPT11+XLP group trended closer to that of the control group (Figure 4B). Circos plot analysis further confirmed differences in dominant microbial communities among the three groups (Figure 4C), highlighting the potential of XLP in modulating gut microbiota composition.

At the family level, the predominant microbiota included *Bacteroidaceae*, *Muribaculaceae*, *Lactobacillaceae*, *Lachnospiraceae*, *Akkermansiaceae*, *Prevotellaceae*, and *Rikenellaceae* (Figure 4D; Table S1, Supporting Information). CPT11 treatment led to a decrease in *Lachnospiraceae* and an increase in *Lactobacillaceae*, whereas XLP administration reversed these trends. Since CPT11‐induced intestinal toxicity is largely attributed to the accumulation of SN38 in the GI tract, and the GUS enzyme plays a key role in converting inactive SN38G into active SN38, we hypothesized that modulating GUS‐producing bacteria might be a primary mechanism by which XLP mitigates CPT11‐induced mucositis. High‐throughput 16S rRNA sequencing of fecal samples revealed a significant increase in several GUS‐producing bacterial genera in the CPT11 group, with *g_Lactobacillus* showing the most pronounced change (Figure 4E). XLP treatment significantly reduced *g_Lactobacillus* abundance, as confirmed by violin plot analysis (Figure 4F) and RT‐qPCR (Figure 4G). Furthermore, a fecal GUS‐generating bacteria detection assay using 4‐methylumbellifery‐β‐D‐glucuronide (4‐MUG) agar demonstrated that XLP directly inhibited the growth of GUS‐producing bacteria (Figure 4H,I).

To determine whether XLP directly inhibits GUS enzyme activity, we assessed fecal lysate supernatants using the PNPG assay. The CPT11 group exhibited a significant increase in GUS activity, which was markedly reduced by XLP treatment (Figure 4J). In vivo imaging with the non‐fluorescent GUS probe FDGlcU further confirmed the inhibitory effect of XLP on GUS enzyme activity (Figure 4K). Molecular docking analysis predicted strong binding affinities between XLP's active compounds and *E. coli* GUS, suggesting that berberine, jateorhizine, coptisine, groenlandicine, magnoflorine, costunolide, and palmatine can interact with the GUS enzyme (Figure S2A, Supporting Information). Notably, coptisine exhibited binding energy comparable to or greater than that of a known GUS inhibitor.^[^ ^28^ ^]^ Enzyme activity assays further demonstrated dose‐dependent GUS inhibition by XLP's active constituents (Figure S2B, Supporting Information), indicating that XLP not only suppresses GUS‐producing bacterial proliferation but also directly inhibits GUS enzyme activity. To further investigate the impact of CPT11 on the GUS [3.2.1.31] gene, we conducted Tax4Fun analysis using 16S rRNA sequencing data, which revealed an increase in GUS gene orthology in the CPT11 group, mitigated by XLP treatment (Figure 4L). RT‐qPCR analysis corroborated these findings, showing that uidA gene mRNA expression, responsible for encoding the GUS enzyme, was elevated in the CPT11 group but significantly reduced by XLP (Figure 4M). As GUS enzyme catalyzes the conversion of SN38G to SN38, a key process driving CPT11‐induced intestinal toxicity,^[^ ^12^ ^]^ we further analyzed fecal CPT11 and SN38 concentrations using LC‐MS/MS. The results demonstrated that XLP treatment significantly reduced intestinal CPT11 and SN38 levels (Figure 4N).

Collectively, these findings indicate that CPT11 disrupts gut microbiota homeostasis, leading to an increased abundance of GUS‐expressing bacteria, particularly *Lactobacillus*, and heightened GUS enzyme activity in the intestinal lumen. In contrast, XLP restores microbial balance by reducing GUS‐producing bacteria, especially *Lactobacillus*, thereby lowering GUS enzyme activity and decreasing SN38 accumulation in the intestinal tract of CPT11‐treated mice. This reduction in intestinal SN38 levels likely contributes to the mitigation of CPT11‐induced intestinal mucositis.

### 2.4 The Transplantation of Dysregulated Gut Microbiota Triggers Intestinal Mucositis, Which is Alleviated by Fecal Microbiota Transplantation (FMT) From XLP‐Treated Mice

**Figure 5:**
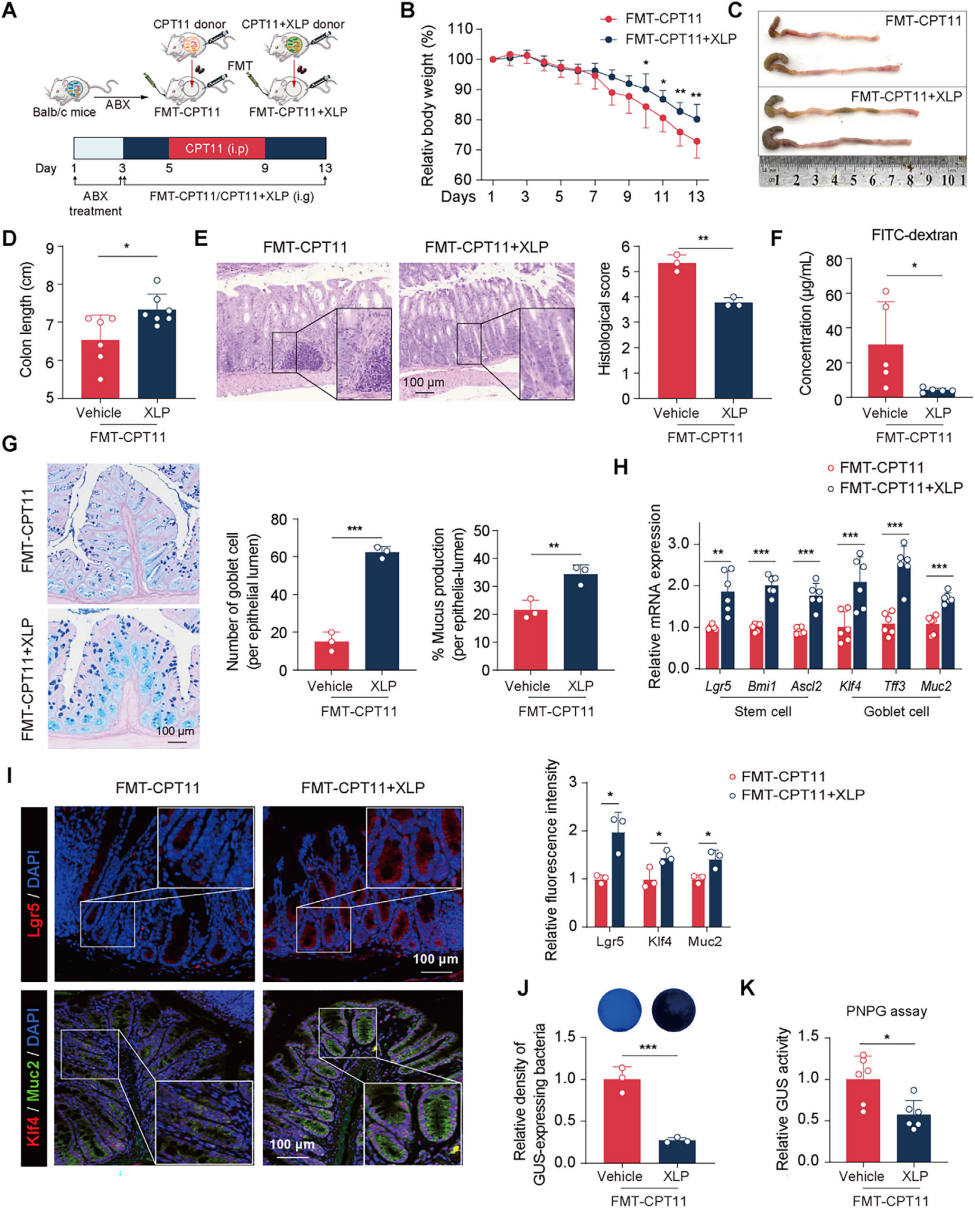
FMT from XLP‐treated donors ameliorated intestinal injury in mucositis mice. A) Experimental design. B) Body weight changes (mean ± SEM, *n* = 6). C, D) Representative colon photographs and length measurements (*n* = 6). E) Colonic sections by H&E staining and histological scoring (*n* = 3). F) Serum concentrations of FITC‐dextran (*n* = 5). G) AB‐PAS staining of colonic sections (scale bar: 100 µm) with quantitative analysis of goblet cells and mucus production (*n* = 3). H) Relative mRNA expressions of cell markers in colon tissues (*n* = 6). I) Immunofluorescence images and quantification of Lgr5 (red), Klf4 (red), and Muc2 (green) in colon tissues (*n* = 3, scale bar: 100 µm). Nuclei labeled with DAPI (blue). J) Images of GUS‐producing bacteria in feces using 4‐MUG supplemented agar, with blue indicating abundance. K) GUS activity in feces detected by PNPG assay (*n* = 4). Data are expressed as Mean ± SD. Statistical analysis was performed using an unpaired Student's *t*‐test (B, D‐K). *p < 0.05, **p < 0.01, ***p < 0.001 versus FMT‐CPT11 group.

To investigate the role of CPT11‐altered microbiota in germ‐free (GF) mice, we performed FMT using microbiota from mice subjected to CPT11‐induced intestinal mucositis with or without XLP treatment. Mice were first treated with a broad‐spectrum antibiotic regimen to eliminate gut microbiota, establishing a GF state. FMT was then conducted, generating two groups: FMT‐CPT11 and FMT‐CPT11+XLP, as illustrated in **Figure** **5**A. FMT‐CPT11 mice exhibited weight loss and persistent diarrhea (Figure 5B). However, starting from day 10, the FMT‐CPT11+XLP group showed a significant reduction in weight loss compared to FMT‐CPT11 mice. Additionally, colon shortening, a hallmark of mucosal inflammation, was markedly alleviated in FMT‐CPT11+XLP mice (Figure 5C,D). Histopathological analysis further revealed substantial improvement in colonic integrity in the FMT‐CPT11+XLP group compared to FMT‐CPT11 mice (Figure 5E). Consistent with these findings, FMT‐CPT11+XLP mice exhibited decreased intestinal permeability, increased goblet cell count, enhanced mucus production, and upregulated expression of ISC markers (Lgr5, Bmi1 (B lymphoma Mo‐MLV insertion region 1), Ascl2 (achaete‐scute family bHLH transcription factor) and goblet cell markers (Klf4, Tff3 (protease‐resistant trefoil factor 3), Muc2) (Figure 5F–H). Furthermore, immunofluorescence staining of colonic samples confirmed a significant increase in ISC stemness markers (Lgr5) and goblet cell differentiation markers (Klf4, Muc2) in FMT‐CPT11+XLP mice compared to FMT‐CPT11 mice (Figure 5I), highlighting the protective role of XLP‐modulated microbiota in maintaining epithelial homeostasis. Additionally, 4‐MUG and PNPG assays revealed a significant reduction in GUS‐producing bacteria (Figure 5J) and lower fecal GUS activity (Figure 5K) in FMT‐CPT11+XLP mice compared to FMT‐CPT11 mice.

To determine whether XLP could mitigate CPT11‐induced intestinal injury in the absence of gut microbiota, we administered broad‐spectrum antibiotics to eliminate gut microbiota in both CPT11‐treated and CPT11+XLP‐treated mice. The effects of XLP on CPT11‐induced enteritis were then reassessed (Figure S3A, Supporting Information). As expected, the antibiotic‐mediated depletion of gut microbiota significantly attenuated the protective effects of XLP, as evidenced by the lack of improvement in CPT11‐induced body weight loss and colon shortening (Figure S3B, C, Supporting Information). Furthermore, no significant differences were observed between the CPT11‐antibiotic (ABX) and CPT11+XLP‐ABX groups in terms of pathological lesions, intestinal permeability, mucus production, and mucus‐secreting goblet cell count (Figure S3D‐F, Supporting Information). These findings suggest that the protective effects of XLP against CPT11‐induced intestinal injury are, to a significant extent, dependent on the presence of an intact gut microbiota.

Collectively, these findings suggest that the dysregulated gut microbiota derived from the feces of CPT11‐treated mice can induce colitis symptoms. Conversely, fecal microbiota from XLP‐treated mice effectively mitigates CPT11‐induced enterotoxicity. Notably, pretreatment with antibiotics diminishes XLP's protective effects, emphasizing that XLP's efficacy in alleviating CPT11‐induced intestinal mucositis relies on gut microbiota composition.

### 2.5 Colonization of GUS‐Expressing *L. Reuteri* Exacerbates CPT11‐Induced Mucositis and Impairs ISC Function, Which is Effectively Alleviated by XLP

**Figure 6:**
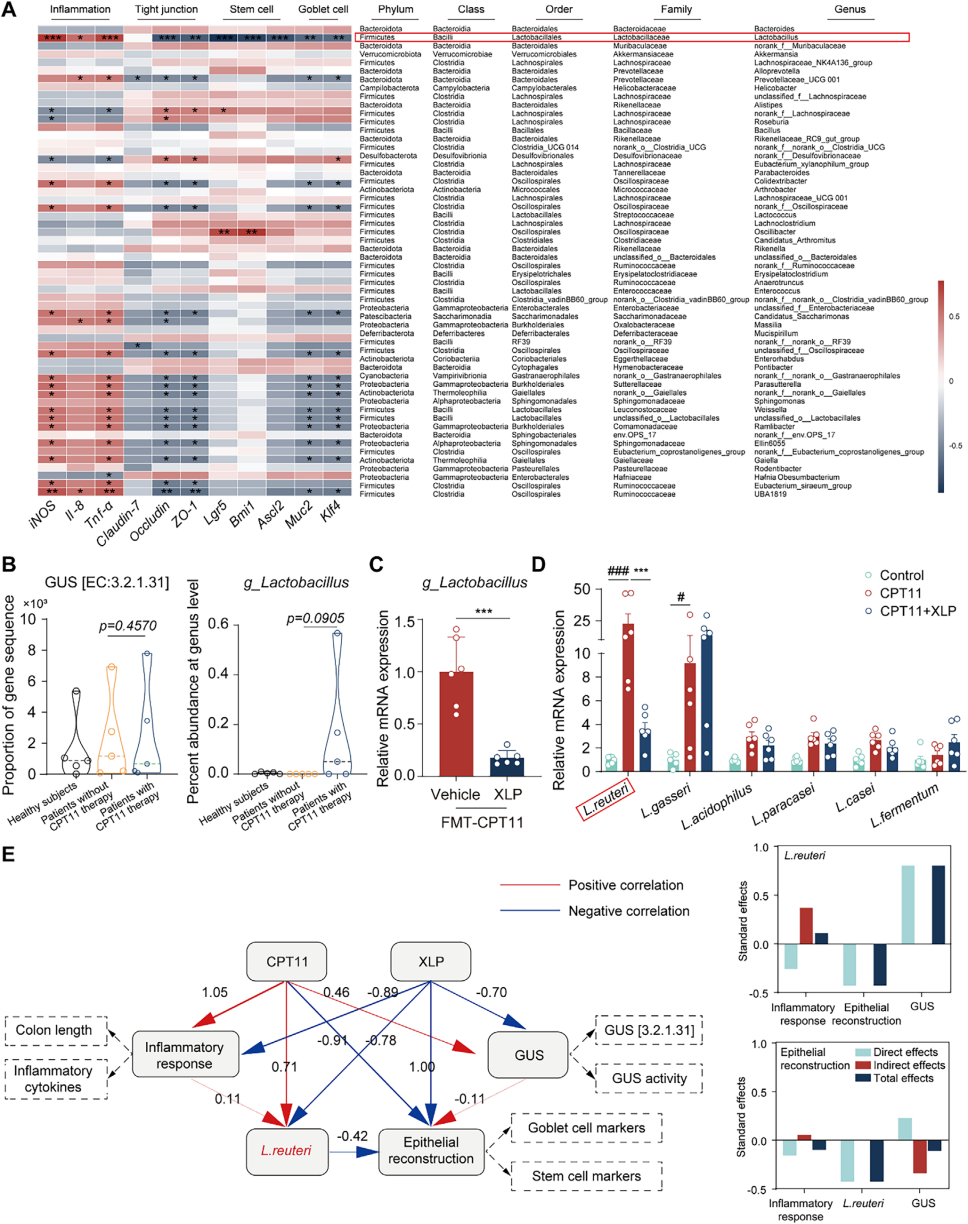
The GUS‐generating bacterial strain *L. reuteri* exhibited a robust correlation with CPT11‐induced enteritis. A) Correlation analysis of gut microbiota indicators. Correlation coefficients: *R* ^2^ > 0.25 (moderate), *R* ^2^ < 0.36 (strong). Significance: *p < 0.05, **p < 0.01, ***p < 0.001. Positive correlations in red, negative in blue, color intensity indicates strength. B) The abundance of ortholog K01195 (GUS) and *g_Lactobacillus* in different groups (*n* = 5), with dotted lines within each violin plot representing the median. C) mRNA levels of *g_Lactobacillus* in feces (*n* = 6). D) mRNA levels of various *Lactobacillus* species in feces (*n* = 6). E) PLS‐PM showing relationships among variables. Path coefficients: red (positive), blue (negative). Model GoF statistic: 0.74. Data are expressed as Mean ± SEM (C, D). Statistical analysis was performed using one‐way ANOVA (B, D) or an unpaired Student's *t*‐test (C). ^###^ *p* < 0.001 versus control group; *p < 0.05, ***p < 0.001 versus CPT11 group.

To investigate the role of key microbial players in CPT11‐induced enterotoxicity, we performed a correlation analysis between gut bacteria and various inflammatory and epithelial markers. The analysis revealed that the genus *Lactobacillus* exhibited a strong positive correlation with pro‐inflammatory markers such as iNOS, IL‐8, and Tnf‐α (**Figure** **6**A). In contrast, *Lactobacillus* showed significant negative correlations with markers of intestinal tight junction integrity (ZO‐1, Claudin‐7, Occludin), crypt stem cells (Lgr5, Bmi1), and goblet cells (Ascl2, Klf4, Muc2). These findings suggest that *Lactobacillus* plays a dual role in modulating intestinal inflammation and epithelial cell function, potentially contributing to CPT11‐induced mucosal injury.

Next, we analyzed fecal 16S rRNA sequencing data from healthy individuals, colorectal cancer patients undergoing CPT11 chemotherapy, and those not receiving CPT11 chemotherapy. The results revealed an increase in fecal GUS abundance (Figure 6B left panel) and a higher abundance of *g_Lactobacillus* in CPT11‐treated patients (Figure 6B right panel), suggesting a potential role of *g_Lactobacillus* in CPT11‐induced GI side effects. To further validate these findings, we assessed *g_Lactobacillus* abundance in FMT murine feces using RT‐qPCR analysis. Consistent with our observations in CPT11+XLP‐treated mice (Figure 4G), *g_Lactobacillus* levels were significantly lower in FMT‐CPT11+XLP mice compared to FMT‐CPT11 mice (Figure 6C). We then examined the relative abundance of key *Lactobacillus* species, including *L. reuteri*, *L. gasseri*, *L. acidophilus*, *L. paracasei*, *L. casei*, and *L. fermentum*, by RT‐qPCR (Figure 6D). Among these species, only *L. reuteri* was significantly elevated in CPT11‐treated mice and subsequently suppressed by XLP administration. Similarly, *L. reuteri* abundance was significantly lower in the FMT‐CPT11+XLP group compared to the FMT‐CPT11 group (Figure S3G, Supporting Information), further supporting the role of *L. reuteri* in CPT11‐induced intestinal toxicity.

To gain a comprehensive understanding of the intricate interactions among key regulatory factors, a Partial Least Squares Path Model (PLS‐PM) was constructed (Figure 6E). This model was designed to elucidate and quantify the relationships between several critical variables, including the GUS‐expressing bacterial abundance (*L. reuteri*), inflammatory response (colon length and inflammatory cytokine levels), epithelial restitution markers (goblet cell and stem cell markers), and GUS metrics (relative GUS [3.2.1.31] abundance and fecal GUS activity). The model revealed that CPT11 exposure was strongly positively correlated with inflammatory response (Pc = 1.05), *L. reuteri* abundance (Pc = 0.71), and GUS metrics (Pc = 0.46), while showing a negative correlation with epithelial restitution (Pc = −0.91) (Figure 6E). In contrast, XLP treatment exhibited negative correlations with inflammatory response (Pc = −0.89), *L. reuteri* abundance (Pc = −0.78), and GUS metrics (Pc = −0.70), while showing a strong positive correlation with epithelial restitution (Pc = 1.00). Notably, *L. reuteri* abundance was negatively correlated with epithelial restitution (Pc = −0.42), suggesting its detrimental effects on intestinal epithelial renewal.

**Figure 7:**
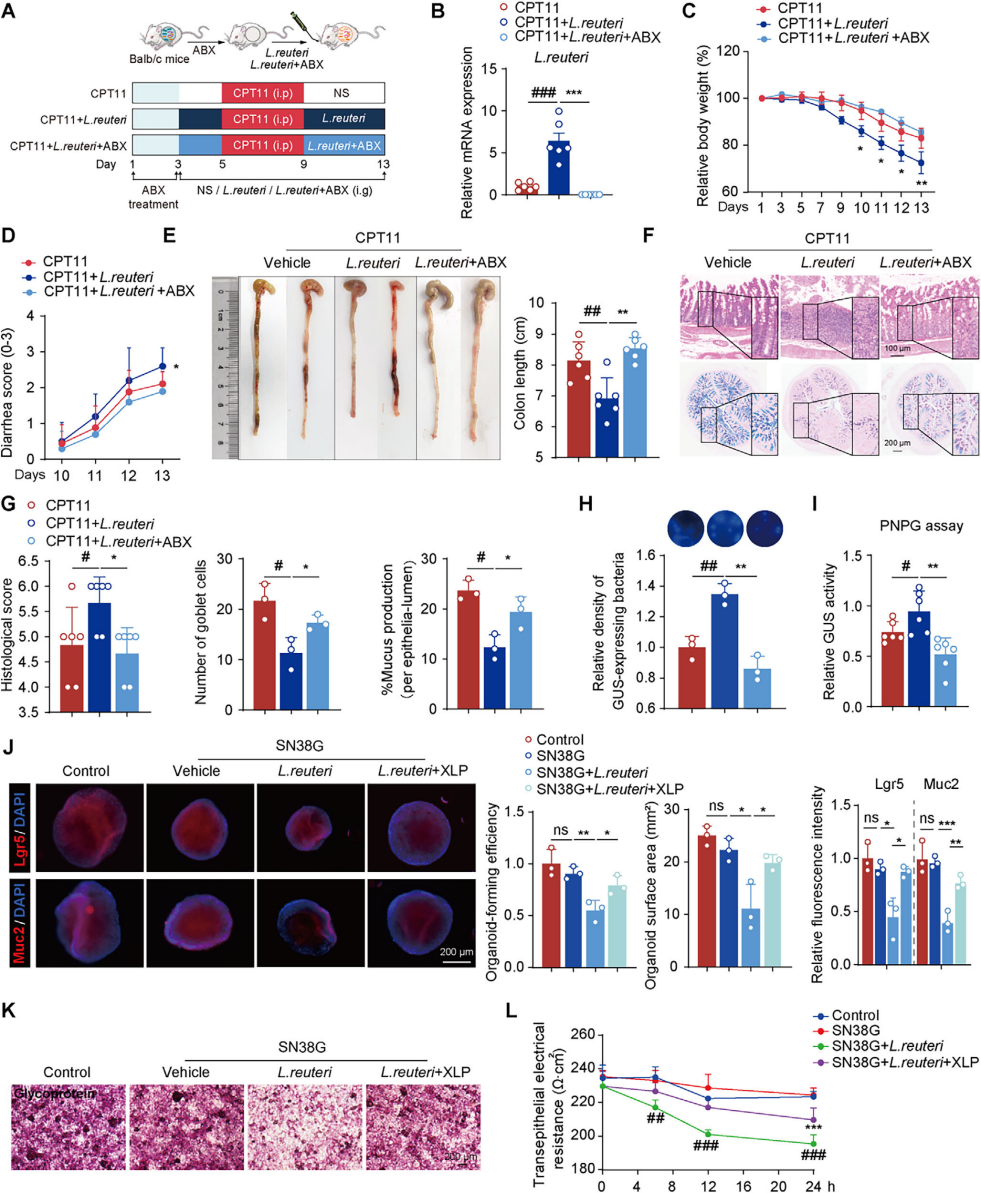
Colonization of *L. reuteri* exacerbated CPT11‐induced enteritis and damaged ISCs. A) Experimental design. B) mRNA levels of *L. reuteri* in feces (*n* = 6). C) Body weight changes (*n* = 6). D) Percentage of mice with bloody diarrhea over time (*n* = 6). E) Representative colon photographs and length measurements (*n* = 6). F) Colonic sections by H&E staining and AB‐PAS staining (scale bar: 100 µm and 200 µm). G) Histological score and analyses of goblet cells number and mucus production. H) Images of GUS‐producing bacteria in feces using 4‐MUG supplemented agar, with blue indicating abundance. I) GUS activity in feces detected by PNPG assay (*n* = 4). J) Colonic organoids treated with various conditions. Bright‐field and immunofluorescence images of Lgr5 and Muc2 (scale bar: 200 µm). Qualitative statistics of organoid‐forming efficiency, surface area, and fluorescence intensity of Lgr5 and Muc2. K) Caco‐2 cell monolayer treated with various conditions; Glycoprotein secretion detected by PAS staining kit. L) NCM460 cell monolayer treated with various conditions; TEER was measured (*n* = 3). Data are expressed as mean ± SEM (C) or mean ± SD (B, D‐E, G‐J, L). Statistical analysis was performed using one‐way ANOVA. ^#^ *p* < 0.05, ^##^ *p* < 0.01, ^###^ *p* < 0.001 versus control group; *p < 0.05, **p < 0.01, ***p < 0.001 versus CPT11 or SN38G group; ns, no significance.

To further investigate the role of *L. reuteri* in CPT11‐induced mucositis, a colonization experiment was conducted (**Figure** **7**A). RT‐qPCR analysis confirmed a significantly elevated bacterial community of *L. reuteri* in the gut of colonized mice, which was notably reduced following antibiotic treatment (Figure 7B). Mice orally administered with *L. reuteri* developed more severe intestinal mucositis than the CPT11 group, exhibiting significant weight loss, diarrhea, colon shortening, reduced goblet cell numbers, and increased pathological damage in colonic tissue (Figure 7C–G). AB‐PAS staining further confirmed the detrimental effect of *L. reuteri* on goblet cells, showing a notable reduction in mucus‐producing cells (Figure 7F, lower panel, and Figure 7G). In contrast, mice treated with antibiotic‐inactivated *L. reuteri* did not experience worsened mucositis compared to the CPT11 group (Figure 7C–G). Further, 4‐MUG and PNPG assays demonstrated a substantial increase in both the population of GUS‐generating bacteria and fecal GUS enzyme activity following *L. reuteri* colonization (Figure 7H,I). However, administering antibiotics to the colonized microbial community almost completely reversed these effects, reinforcing the role of *L. reuteri* in exacerbating CPT11‐induced mucositis via its GUS activity.

Additionally, in vitro treatment of *L. reuteri* with XLP or its major active components (berberine, coptisine, palmatine, jateorhizine, and costunolide) significantly suppressed *L. reuteri* proliferation in a time‐ and dose‐dependent manner (Figure S4A, Supporting Information). A real‐time monitoring system further provided a visual representation of XLP's inhibitory effect on GUS enzyme‐producing bacteria (Figure S4B, Supporting Information). Since SN38 is enzymatically converted from SN38G by GUS‐producing bacteria, leading to direct mucosal toxicity, we further examined its impact using a colonic organoid model. Co‐incubation of organoids with SN38G and *L. reuteri* resulted in a reduced organoid‐forming efficiency and surface area, along with decreased expression of Lgr5 and Muc2 (Figure 7J). However, XLP treatment effectively mitigated these adverse effects, restoring organoid‐forming efficiency, surface area, and the expression of marker genes (Lgr5, Muc2). To further evaluate these effects, we utilized a Caco‐2 cell model, in which co‐incubation with SN38G and *L. reuteri* led to reduced mucosal polysaccharide expression, which was restored by XLP treatment (Figure 7K). Similarly, TEER assays demonstrated that XLP effectively restored mucosal integrity disrupted by *L. reuteri* (Figure 7L).

Overall, these findings indicate that GUS‐expressing *L. reuteri* significantly worsens CPT11‐induced intestinal mucositis. However, XLP effectively alleviates CPT11‐induced enterotoxicity by suppressing *L. reuteri*, thereby reducing GUS enzyme activity and limiting the conversion of SN38G to SN38 in the intestinal lumen. This protective effect prevents damage to intestinal crypt structures and preserves intestinal barrier integrity, indirectly promoting ISC proliferation and differentiation, which facilitates mucosal repair following CPT11‐induced injury.

### 2.6 XLP Exhibited a Synergistic Anti‐Tumor Effect with CPT11

Given that XLP reduces intestinal SN38 levels, we further assessed its impact on CPT11's anticancer efficacy. In a mouse colorectal cancer xenograft model, co‐treatment with XLP (0.5 or 1 g kg^−1^) and CPT11 significantly delayed tumor growth compared to CPT11 alone (Figure S5A‐D, Supporting Information). In vitro CCK‐8 assays demonstrated that XLP and its major active compounds (berberine, palmatine, jateorhizine, coptisine, and costunolide) dose‐dependently suppressed the proliferation of CT26 colon cancer cells (Figure S5E, F, Supporting Information). Furthermore, Combination Index (CI) analysis confirmed the synergistic inhibitory effects of XLP and its active compounds when combined with CPT11, further supporting its potential as an adjuvant therapy (Table S2, Supporting Information).

These findings suggest that XLP effectively mitigates CPT11‐induced intestinal toxicity while preserving and even enhancing CPT11's anticancer efficacy through synergistic effects, highlighting its potential as a complementary therapeutic strategy.

## 3 Discussion

In the present study, we demonstrated that chemotherapy with CPT11 significantly impacts the gut microbiota, leading to dysbiosis characterized by an increased population of GUS‐expressing bacteria. This shift in microbial composition has a detrimental influence, as GUS enzyme generated from GUS‐expressing bacteria converts the non‐toxic metabolite SN38G to the toxic SN38, which damages the regenerative epithelial stem/progenitor cell pool and exacerbates CPT11‐induced mucosal injury. The selective suppression of GUS‐expressing bacteria without the use of broad‐spectrum antibiotics can effectively mitigate these adverse effects, highlighting the benefits of targeted microbial modulation in managing chemotherapy‐induced GI adverse effects.

To investigate the role of key microbial players in CPT11‐induced enterotoxicity, we performed a correlation analysis between gut bacteria and various inflammatory and epithelial integrity markers. Notably, we identified the genus *Lactobacillus*, particularly *L. reuteri*, as having the strongest positive correlations with pro‐inflammatory markers such as iNOS, IL‐8, and TNF‐α. Conversely, *L. reuteri* exhibited notable negative correlations with markers of intestinal tight junction integrity (ZO‐1, Claudin‐7, Occludin), ISC cells (Lgr5, Bmi1), and goblet cells (Ascl2, Klf4, Muc2). These correlations suggest that *L. reuteri* plays a dual role in modulating intestinal inflammation and epithelial function, potentially contributing to CPT11‐induced mucosal injury. Further analysis of fecal 16S rRNA sequencing data from colorectal cancer patients undergoing CPT11 chemotherapy revealed an increase in fecal GUS abundance and *Lactobacillus* levels, particularly *L. reuteri*. This finding supports the hypothesis that *L. reuteri* is a key player in the gut microbiota's response to CPT11 chemotherapy and may contribute to its intestinal toxicity.

To directly assess the impact of *L. reuteri* on CPT11‐induced mucositis, we conducted a colonization experiment by introducing *L. reuteri* into the gut of mice. The introduction of *L. reuteri* significantly worsened intestinal damage caused by CPT11, as evidenced by increased weight loss, diarrhea, and colon shortening in colonized mice. Histopathological analysis further revealed severe epithelial damage and a reduction in goblet cell numbers in the colons of *L. reuteri*‐colonized mice. AB‐PAS staining confirmed these findings, showing decreased mucus production, reinforcing the detrimental effects of *L. reuteri* on goblet cells. In contrast, mice administered with antibiotic‐treated *L. reuteri* did not experience worsened mucositis compared to the CPT11 group, suggesting that the harmful effects of *L. reuteri* are primarily mediated through its GUS activity. Further 4‐MUG and PNPG assays revealed a substantial increase in both GUS‐producing bacteria and GUS enzyme activity in the feces of *L. reuteri*‐colonized mice, indicating that *L. reuteri* enhances the conversion of SN38G to toxic SN38, thereby exacerbating mucosal injury. Notably, administering antibiotics to the colonized microbial community nearly completely reversed these effects, further confirming the key role of *L. reuteri* in mediating CPT11‐induced mucositis.

To investigate the protective effects of XLP, we treated *L. reuteri* with XLP or its major active components (berberine, coptisine, palmatine, jateorhizine, and costunolide). These treatments inhibited *L. reuteri* proliferation in a time‐ and dose‐dependent manner. A real‐time monitoring system provided an intuitive visualization of XLP's inhibitory effect on GUS enzyme‐producing bacteria. Moreover, incubation of colonic organoids with *L. reuteri* and SN38G resulted in reduced organoid‐forming efficiency and surface area, along with decreased expression of ISC marker Lgr5 and goblet cell marker Muc2. However, XLP treatment effectively mitigated these adverse effects, restoring organoid‐forming efficiency, surface area, and ISC marker gene expression. Further analysis using a Caco‐2 intestinal epithelial cell model revealed that co‐incubation with SN38G and *L. reuteri* led to a reduction in mucosal polysaccharide expression, which was restored by subsequent XLP treatment. Similarly, TEER assays demonstrated that XLP restored mucosal integrity disrupted by *L. reuteri*, reinforcing the effects of XLP in protecting the intestinal barrier.

Overall, these findings demonstrate that the GUS‐expressing bacterium *L. reuteri* significantly exacerbates CPT11‐induced intestinal mucositis by enhancing the conversion of SN38G to SN38, leading to increased mucosal damage. However, XLP treatment effectively mitigates these effects by suppressing *L. reuteri* proliferation and its GUS enzyme activity. This suppression protects ISCs and enables rapid reconstitution of the injured epithelium by fostering ISC renewal and differentiation into mucus‐secreting goblet cells, thereby fortifying the mucosal barrier. These results underscore the potential of targeting specific gut microbiota, such as *L. reuteri*, as a therapeutic strategy to promote mucosal healing and alleviate chemotherapy‐induced GI toxicity.

Notably, *L. reuteri*, a gram‐positive bacterium and a natural member of the human gut microbiota, has attracted attention for its health‐promoting benefits.^[^ ^29^ ^]^ Extensive research suggests that *L. reuteri* enhances gut motility, combats pathogenic infections, and strengthens intestinal barrier function.^[^ ^30^ ^]^ However, despite these beneficial effects, *L. reuteri* has also been associated with increased susceptibility to autoimmune diseases.^[^ ^31^ ^]^ For instance, mice co‐colonized with *L. reuteri* and another probiotic species exhibited greater susceptibility to autoimmune encephalomyelitis symptoms.^[^ ^31^ ^]^ Our findings align with previous studies implicating *L. reuteri* and its GUS enzyme in mucositis pathogenesis. By directly inhibiting the growth of GUS‐expressing *L. reuteri*, XLP reduced intestinal GUS levels and SN38 accumulation, indirectly enhancing ISC proliferation and differentiation to repair CPT11‐induced mucosal damage. These results underscore the potential of targeting specific gut microbiota, such as *L. reuteri*, as a therapeutic strategy to promote mucosal healing and mitigate chemotherapy‐induced GI toxicity.

An important consideration is whether reducing intestinal SN38 levels might compromise CPT11's anticancer efficacy. Interestingly, our findings indicate that XLP not only alleviates CPT11‐induced GI toxicity but also enhances its antitumor activity. In a mouse colorectal cancer xenograft model, co‐treatment with XLP and CPT11 significantly delayed tumor growth compared to CPT11 alone, demonstrating a synergistic effect characterized by improved overall health and reduced tumor burden in treated mice. In vitro assays further confirmed these findings. The CCK‐8 assay demonstrated that XLP and its major active compounds (berberine, palmatine, jateorhizine, coptisine, and costunolide) dose‐dependently suppressed the proliferation of CT26 colon cancer cells. Moreover, Combination Index (CI) analysis confirmed the synergistic inhibitory effects of XLP and its active compounds with CPT11, indicating that the combined treatment was more effective than either agent alone. These results suggest that bacterial GUS inhibition selectively protects the intestinal epithelium while preserving SN38 levels and cytotoxicity within the tumor microenvironment. Supporting this notion, our previous study demonstrated that berberine, a major bioactive component of XLP, enhances CPT11's anticancer efficacy while simultaneously suppressing intestinal GUS enzyme activity.^[^ ^32^ ^]^

Unlike previous studies that primarily focused on XLP's broad anti‐inflammatory and microbiota‐modulating properties,^[^ ^23^, ^33^, ^34^ ^]^ our findings offer a distinct perspective by highlighting the critical role of GUS‐expressing bacteria, particularly *L. reuteri*, in mediating CPT11‐induced intestinal toxicity. Specifically, we found that CPT11 chemotherapy disrupts gut homeostasis by increasing the abundance of *L. reuteri*, a key GUS‐expressing bacterial strain, which in turn enhances the conversion of SN38G into SN38. This process exacerbates mucosal injury and depletes the intestinal regenerative system. XLP mitigates these adverse effects not only by reducing inflammation but also by directly suppressing *L. reuteri* proliferation and its GUS enzymatic activity, thereby lowering luminal SN38 accumulation and protecting ISCs. Furthermore, our study establishes a mechanistic link between XLP's microbiota‐modulating effects and intestinal epithelial regeneration, a connection that was not explicitly explored in previous research. By inhibiting *L. reuteri*, XLP preserves ISC function and promotes differentiation into secretory‐lineage cells, thereby restoring mucosal integrity and strengthening the intestinal barrier. Interestingly, studies have shown that Xiao‐Chai‐Hu‐Tang (XCHT), a well‐known traditional Chinese medicine, effectively inhibits GUS enzymatic activity, reducing the conversion of SN38G to SN38 and thereby mitigating CPT11‐induced mucosal injury.^[^ ^35^ ^]^ Our study identifies XLP as a microbiota‐modulating agent that selectively suppresses GUS‐producing bacteria, particularly *L. reuteri*, while simultaneously preserving the crypt stem cell niche. Moreover, our data reveal that XLP not only reduces CPT11‐induced side effects but also enhances its antitumor activity through a synergistic mechanism, highlighting its dual therapeutic potential.

While the current study provides compelling evidence for the role of GUS‐expressing bacteria in CPT11‐induced enterotoxicity and highlights the therapeutic potential of XLP in mitigating CPT11‐induced intestinal mucositis without compromising CPT11's anticancer efficacy, several limitations warrant further investigation. First, the long‐term effects of XLP on gut microbiota composition and overall health remain unclear and require further research. Despite this, our findings provide a strong rationale for clinical evaluation of XLP as a microbiome‐modulating adjuvant to alleviate dose‐limiting intestinal toxicity associated with CPT11 chemotherapy. Second, although murine models offer valuable mechanistic insights, their translatability to humans needs validation through clinical trials. Future studies should focus on refining therapeutic strategies, including the development of more specific GUS inhibitors, the exploration of probiotics or microbiota‐targeted therapies that could synergize with chemotherapy, and a deeper investigation into the molecular interactions between gut microbiota and host cells to guide the design of more effective interventions. Currently, the management of CPT11‐induced diarrhea and mucositis primarily relies on antidiarrheal agents and anti‐inflammatory drugs, which provide only symptomatic relief and may lead to complications such as antibiotic resistance and intestinal obstruction. In contrast, our study proposes a paradigm shift toward a microbiota‐targeted approach, specifically targeting GUS‐expressing bacteria as a therapeutic strategy. By preserving ISC function and promoting epithelial regeneration, this approach addresses the root cause of chemotoxicity rather than merely alleviating symptoms, offering a more effective and sustainable solution for managing chemotherapy‐induced GI toxicity.

## 4 Conclusion

Our study demonstrates that GUS‐expressing *L. reuteri* plays a pivotal role in mediating CPT11 chemotherapy‐induced intestinal injury and depleting the regenerative epithelial stem/progenitor pool. The antidiarrheal agent XLP effectively mitigates CPT11‐induced mucositis by suppressing *L. reuteri* proliferation and reducing its GUS activity, thereby lowering luminal SN38 accumulation and protecting the intestinal epithelium. This, in turn, preserves the mucosal stem cell niche, facilitating the rapid regeneration of secretory lineages that strengthen the epithelial barrier. Targeting the “GUS microbe‐host‐irinotecan axis” represents a promising microbiome‐based strategy to counteract chemotherapy‐induced GI toxicity. Notably, selective suppression of GUS‐expressing bacteria without the use of broad‐spectrum antibiotics can effectively alleviate CPT11‐induced toxicity by promoting ISC proliferation, renewal, and differentiation. These findings highlight the therapeutic potential of precise microbial modulation in managing chemotherapy‐induced GI side effects while maintaining overall gut homeostasis.

## 5 Experimental Section

### Materials

Irinotecan (CPT11, Chemical Abstracts Service (CAS) number: 136572‐09‐3; molecular weight (MW): 677.18), SN38 (CAS: 86639‐52‐3; MW: 392.40), ampicillin (CAS: 7177‐48‐2; MW: 403.45), neomycin (CAS: 1405‐10‐3; MW: 908.87), vancomycin (CAS: 1404‐93‐9; MW: 1485.72), metronidazole (CAS: 443‐48‐1; MW: 171.15), berberine (CAS: 633‐65‐8; MW: 371.81), palmatine (CAS: 10605‐02‐4; MW: 387.86), jateorhizine (CAS: 960383‐96‐4; MW: 373.83), coptisine (CAS: 6020‐18‐4; MW: 355.77), and dehydrocostus (CAS: 477‐43‐0; MW: 230.30) were purchased from Dalian Meilun Biotech Co., Ltd. (Dalian, China). Costunolactone (CAS: 553‐21‐9; molecular weight: 232.32) was purchased from Chengdu Purifay technology Co., Ltd (Chengdu, China). XLP was purchased from Nodse Pharmacy (Hubei, China). The following antibodies were used: iNOS (catalog number: 18985‐1‐AP; Proteintech, Chicago, IL, USA), COX‐2 (12375‐1‐AP; Proteintech), Claudin‐7 (10118‐1‐AP; Proteintech), ZO‐1 (A11417; ABclonal Technology, Woburn, MA, USA), Occludin (A2601; ABclonal Technology), Klf4 (A13673; ABclonal Technology), MUC2 (A4767; ABclonal Technology), and β‐actin (4970; Cell Signaling Technology, Danvers, MA, USA). FITC‐dextran was obtained from Sigma (CAS: 60842‐46‐8). Diethylpyrocarbonate‐treated water and dimethyl sulfoxide were obtained from MilliporeSigma (Burlington, MA, USA). Enhanced chemiluminescence (ECL) detection kit was obtained from Millipore (Billerica, MA, USA). TRIzol Reagent and the SuperScript II Reverse Transcriptase kit were purchased from Thermo Scientific (Waltham, MA, USA). SYBR Premix ExTaq Mix was sourced from Takara Biotechnology (Shiga, Japan). Glutaraldehyde (2.5%; P1126) was obtained from Solarbio Life Science (Beijing, China). PNPG (CAS: 2492‐87‐7) was obtained from MedChemExpress (Monmouth Junction, NJ, USA) and 4‐MUG (CAS: 6160‐80‐1) was from Shanghai YuanYe Biotechnology (Shanghai, China).

### Animals

6‐week‐old male Balb/c mice were housed in the Laboratory Animal Center at Shanghai University of Traditional Chinese Medicine (SHUTCM, Shanghai, China). All procedures were performed according to the guiding principles of the declaration and recommendations of the Animal Experimentation Ethics Committee of SHUTCM. The study was conducted according to the declaration and recommendations of the Animal Experimentation Ethics Committee of Shanghai University of Traditional Chinese Medicine. All animal experiments were approved by the institutional ethics committees of Shanghai University of Traditional Chinese Medicine (Approval Number: PZSHUTCM200828001, PZSHUTCM200911012, PZSHUTCM200724006).

### Establishment of a CPT11‐Induced Intestinal Mucositis Model in Colorectal Cancer Xenograft Mice

The animal model was established following a previously described method.^[^ ^32^ ^]^ CT26 murine colon carcinoma cells (American Type Culture Collection, ATCC, Manassas, VA, USA) were cultured in Roswell Park Memorial Institute (RPMI) 1640 medium (Gibco BRL, NY, USA), supplemented with 10% fetal bovine serum (Gibco BRL, NY, USA). A suspension of 1×10^6^ CT26 cells was subcutaneously injected into the right flank of mice (Figure 1A). Three days later, tumor‐bearing mice were randomly divided into four groups (*n* = 6–8 per group). The control group received 0.5% sodium carboxymethylcellulose (CMC‐Na) orally from days 3 to 14. The other three groups received intraperitoneal injections of CPT11 (60 mg kg^−1^) once daily from days 5 to 9, following a previously established protocol.^[^ ^36^ ^]^ Additionally, two of these groups received oral XLP (0.5 or 1.0 g kg^−1^) treatment once daily from days 3 to 14. Body weight was recorded daily, and all mice were sacrificed on day 14. Blood, colons, and tumors were collected for analysis, and histological damage was scored as described previously.^[^ ^32^, ^36^ ^]^

### FMT Experiment

Mice were randomly divided into four groups: CPT11 donor (*n* = 4), CPT11+XLP donor (*n* = 4), FMT‐CPT11 (*n* = 10), and FMT‐CPT11+XLP (*n* = 10) (Figure 5A). Before FMT, all mice underwent a 6‐hour fast, followed by oral administration of 0.2 mL of an antibiotic cocktail containing 0.5 g L^−1^ neomycin, 0.5 g L^−1^ vancomycin, 1 g L^−1^ ampicillin, and 1 g L^−1^ metronidazole for three consecutive days. For FMT, fresh feces (50 mg) from CPT11 or CPT11+XLP donors were homogenized in 1 mL sterile PBS and centrifuged at 500 g for 1 min. Mice were then orally administered 200 µL of the supernatant once daily. CPT11 (60 mg kg^−1^) was intraperitoneally injected once daily, beginning two days after FMT.

### Antibiotic‐Treated Pseudo‐Sterile Mouse Experiment

To deplete gut microbiota, mice received oral gavage of 0.2 mL of an “ABX cocktail” for three consecutive days (Figure S3A, Supporting Information). Following antibiotic treatment, mice were randomly divided into two groups: CPT11‐ABX (*n* = 6) and CPT11+XLP‐ABX (*n* = 6). Both groups received intraperitoneal CPT11 injections (60 mg kg^−1^) once daily from days 3 to 9. Additionally, mice in the CPT11+XLP‐ABX group were orally administered XLP (1 g kg^−1^) from days 3 to 13. To maintain microbiota depletion, both groups continued receiving the “ABX cocktail” in their drinking water from days 3 to 13.

### Bacterial Colonization Experiment

Mice were randomly divided into three groups (*n* = 6 per group) (Figure 7A). *L. reuteri* (ATCC23272) were obtained from Ning Bo Testobio Co., Ltd (Ningbo, China) and cultured in MRS medium (Coolaber Science & Technology, Beijing, China). Bacterial growth was monitored by measuring optical density at 600 nm, and colony‐forming units (CFU) were determined. Initially, all groups received oral gavage of 0.2 mL ABX cocktail for three consecutive days to deplete gut microbiota. From days 3 to 9, mice were treated with intraperitoneal CPT11 injections (60 mg kg^−1^) once daily. The CPT11 + *L. reuteri* group received daily oral gavage of 10⁸ CFU *L. reuteri* suspended in 200 µL saline from days 1 to 13, while the CPT11 + *L. reuteri* + ABX group followed the same regimen but received *L. reuteri* pre‐treated with ABX before administration. Throughout the experiment, behavioral patterns were routinely monitored, and body weight was systematically recorded for all mice.

### Intestinal Organoid Isolation and Culture

Colon crypts were isolated from C57BL/6 mice following the protocol described in the previous study.^[^ ^36^, ^37^ ^]^ The isolated crypts were suspended in Matrigel (Corning, NY, USA) and seeded into 24‐well plates, then cultured in an incubator at 37 °C with 5% CO₂. IntestCult OGM Mouse Basal Medium (Stemcell Technologies, CA, USA) was added to the wells and refreshed every other day to maintain organoid growth and viability.

### Histological Analysis of Colon

Colon samples were fixed in 4% formalin at ambient temperature, embedded in paraffin, and sectioned at 5 µm thickness. The sections were stained with haematoxylin and eosin (H&E) for general histopathological assessment or subjected to Alcian blue (AB) and Periodic Acid‐Schiff (PAS) staining to evaluate mucin levels and goblet cell counts. Histopathological features were observed under a light microscope and scored according to previously described criteria.^[^ ^32^, ^36^ ^]^

### Immunofluorescence Staining

Colon tissues were fixed with 4% paraformaldehyde (PFA) and sequentially dehydrated with 15% and 30% sucrose in PBS. Frozen tissue sections were dewaxed and incubated with primary antibodies (1:200 dilution), including rabbit anti‐ZO‐1, mouse anti‐Muc2, rabbit anti‐Klf4, and rabbit anti‐Lgr5. Secondary antibodies were ABflo 594‐conjugated Goat Anti‐Rabbit IgG (H+L) and ABflo 488‐conjugated Goat Anti‐Mouse IgG (H+L). Nuclei were counterstained with DAPI for 10 min, and images were captured using an Olympus IX73 microscope (Tokyo, Japan).

### Intestinal Permeability Detection

Intestinal permeability was assessed following a previously described protocol.^[^ ^32^ ^]^ Briefly, mice were orally administered FITC‐dextran at a dose of 40 mg per 100 g body weight. Serum samples were then collected, and fluorescence intensity was measured using fluorometry with an excitation wavelength of 485 nm and an emission wavelength of 528 nm.

### Transmission Electron Microscopy Analysis

Colon tissues from control, CPT11, and CPT11+XLP mice were harvested and fixed with 2.5% glutaraldehyde in 0.1 M sodium cacodylate buffer (pH 7.2) containing 0.1% CaCl₂ for 3 h after PBS washing. Tissues were then post‐fixed for 2 h in 1% osmium tetroxide with 0.1% CaCl₂ in 0.1 M sodium cacodylate buffer. After rinsing with cold ddH₂O, samples were gradually dehydrated in ethanol and propylene oxide at 4 °C and embedded in Embed‐812 (Thermo Fisher Scientific, 14 120), followed by polymerization at 60 °C for 36 h. Ultrathin sections (70–80 nm) were prepared using a diamond knife and ultramicrotome (Ultracut UC7; Leica, Wetzlar, Germany). Sections were stained with methylene blue, followed by 1% uranyl acetate and lead citrate staining. Images were captured using a Bio‐High Voltage EM system (JEM1400 Plus and JEM‐1000 BEF; Jeol, Tokyo, Japan).

### Enzyme Linked Immunosorbent Assay (ELISA)

Blood serum samples were collected to measure LPS and DAO levels using ELISA kits (Yingxin Laboratory Co., Ltd, Shanghai, China), following the manufacturer's instructions.

### Transcriptomic Analysis

Colonic tissue RNA samples were extracted using TRIzol Reagent, and RNA quantity and quality were assessed using a Nanodrop‐2000 spectrophotometer. RNA integrity was verified by electrophoresis, and the RNA integrity number (RIN) was determined using a Bioanalyzer (2100 series; Agilent Technologies, Santa Clara, CA, USA). Evaluation criteria included total RNA content ≥1 µg, concentration ≥35 ng/µL, A₂₆₀/A₂₈₀ ≥ 1.8, and A₂₆₀/A₂₃₀ ≥ 1.0. mRNA isolated from total RNA was sequenced using the HiSeq 2000 platform by Majorbio BioPharm Technology (Shanghai, China).

### 16S rRNA Gene Sequencing

Genomic DNA was extracted from fecal samples using the E.Z.N.A. Soil DNA Kit (Omega Bio‐tek, Norcross, GA, USA) following the manufacturer's protocols. DNA quality was assessed using a NanoDrop 2000 spectrophotometer (Thermo Scientific). The bacterial 16S rRNA gene V3‐V4 region was then amplified using a PCR system (ABI‐GeneAmp‐9700; Applied Biosystems, Carlsbad, CA, USA). The experimental procedure for 16S rRNA sequencing followed the protocol described in the previous study.^[^ ^36^ ^]^ Sequencing was performed on the MiSeq platform (Illumina, San Diego, CA, USA) according to the standard guidelines of Majorbio BioPharm Technology. Microbial community stability was assessed using the AVD metric, which quantifies deviations in the mean relative abundance of taxa at the genus level from a normal distribution, where lower AVD values indicate greater stability. Principal Coordinates Analysis (PCoA) was conducted using Bray‐Curtis distance metrics to evaluate microbial community structure shifts and determine sample similarity or dissimilarity. The distribution and relative abundance of dominant species, as well as their variability across different groups, were visualized using Circos plots.

### Cell Viability Assay

CT26 murine colon carcinoma cells were seeded into 96‐well plates and treated with varying concentrations of XLP (50, 75, 100, 150 µg mL^−1^) or its active compounds, including berberine, palmatine, jateorhizine, coptisine, and costunolide (25, 50 µM each). XLP used in all in vitro experiments was prepared by extraction with a hydrochloric acid:methanol (1:100) mixture, followed by concentration to obtain a dried product. After 24 h of treatment, the culture medium was replaced with fresh medium containing 10% CCK‐8 reagent (Dojindo, Kumamoto, Japan). Following 30 min of incubation in the dark at 37 °C, optical density (OD) at 450 nm was measured using a microplate reader. Relative cell viability (%) was calculated using the formula: [OD (treated) – OD (blank)] / [OD (control) – OD (blank)] × 100%.

### Transepithelial Electrical Resistance (TEER) Assay

NCM460 human normal intestinal epithelial cells (ATCC, Manassas, VA, USA) were cultured in RPMI 1640 medium supplemented with 10% fetal bovine serum (Gibco BRL, NY, USA). Cells were seeded at a density of 5 × 10⁴ cells per 400 µL per well into 24‐well plates with Millicell culture inserts, with 600 µL of RPMI 1640 basal medium added outside the inserts. The culture medium was refreshed every other day, and TEER was measured using an MERS00002 ohmmeter. Once the TEER value reached 200 Ω cm^2^, indicating monolayer integrity, treatments were applied, including SN38 (500 nM), XLP (50 µg mL^−1^), or a combination of SN38 (500 nM) + XLP (50 µg mL^−1^). Following treatment, TEER measurements were taken at 0, 6, 12, and 24 h to assess changes in epithelial barrier integrity.

### Scratch Healing Assay

The migration of NCM460 cells was evaluated using an ibidi insert‐based scratch assay. Cells were seeded into inserts at a concentration of 5 × 10⁴ cells mL^−1^ with 70 µL per chamber. Once confluent, the inserts were carefully removed to create a scratch wound. Cells were then exposed to serum‐free medium containing XLP (50 µg mL^−1^), SN38 (500 nM), or a combination of SN38 (500 nM) + XLP (50 µg mL^−1^). Images of the scratch area were captured at 0 and 24 h post‐treatment using an inverted microscope, and the scratch width was quantified using ImageJ software. Cell migration distance (%) was calculated as: [width (0 h, treated) – width (24 h, treated)] / [width (0 h, control) – width (24 h, control)] × 100%.

### Cellular PAS Staining

Caco‐2 human colonic adenocarcinoma cells (ATCC, Manassas, VA, USA) were cultured in modified Eagle's medium (MEM; Gibco BRL, NY, USA) and seeded into 24‐well plates. Cells were divided into four groups: control, SN38G, SN38G + *L. reuteri*, and SN38G + *L. reuteri* + XLP. After 24 h of treatment, cells were fixed with PAS fixative for 15 min, followed by incubation with an oxidizing agent for another 15 min. The cells were then stained with Schiff reagent and counterstained with Mayer's hematoxylin for 1.5 min. Following thorough washing with water, stained cells were examined and imaged under an inverted microscope.

### Real‐Time Fluorescence Quantitative Polymerase Chain Reaction (RT‐qPCR)

Total RNA was extracted using TRIzol reagent and reverse‐transcribed into cDNA using the Evo M‐MLV RT Premix for qPCR kit. RT‐qPCR reactions were conducted on an ABI Prism 7900HT Sequence Detection System (Life Technologies) with a SYBR Green Premix × Pro Taq HS qPCR Kit. Primer sequences are provided in Table S3 (Supporting Information). Gene expression levels were normalized to β‐actin.

### Western Blotting

Colon segments were homogenized in radioimmunoprecipitation assay (RIPA) buffer supplemented with protease and phosphatase inhibitor cocktail tablets to extract proteins. The extracted proteins were denatured by boiling for 10 min and subsequently separated by SDS‐PAGE (30 µg per lane) before being transferred onto polyvinylidene fluoride (PVDF) membranes for immunoblotting. Membranes were blocked with 5% (w/v) skimmed milk in PBST for 2 h, followed by overnight incubation at 4 °C with specific primary antibodies. After PBST washing, membranes were incubated with corresponding secondary antibodies for 1 h. Protein bands were visualized using enhanced chemiluminescence (ECL) detection reagents. The following primary antibodies were used (1:1000 dilution each): Cox‐2, iNOS, Claudin‐7, ZO‐1, Occludin, Klf4, Muc2, Lgr5, and β‐actin.

### Liquid Chromatography‐Tandem Mass Spectrometry (LC‐MS/MS) Analysis

The quantification of CPT11 and SN38 (Meilunbio, Dalian, China) was performed using LC‐MS/MS, following the protocols described in the previous study.^[^ ^32^ ^]^ Briefly, 10 µL of each sample was injected into the Agilent 1100 LC/MS system equipped with an Acquity UPLC HSS T3 column (2.1 × 100 mm, 1.8 µm). Gradient elution was conducted at a flow rate of 0.4 mL min^−1^ using a mobile phase consisting of 0.1% formic acid in water (solvent A) and 100% acetonitrile (solvent B). The gradient program was as follows: 0–0.5 min, 30% B → 75% B; 0.5‐6 min, 75% B → 80% B; 6–7 min, 80% B → 90% B; 7.01‐10 min, 30% B (re‐equilibration).

### Antibacterial Activity Assay

*L. reuteri* was cultured in MRS medium until reaching the logarithmic growth phase. To evaluate the antibacterial effects of XLP and its active compounds, the bacterial density was adjusted to OD600 = 0.1. XLP was administered at final concentrations of 0, 100, 200, and 400 µg mL^−1^, while its major active compounds (berberine, palmatine, jateorhizine, coptisine, and costunolide) were added at final concentrations of 0, 50, 100, and 200 µg mL^−1^. Antimicrobial activity was assessed using the 96‐well plate OD determination method, with OD600 values measured at 0, 3, 6, and 12 h.

### GUS Enzyme Activity Assays

To visualize intestinal GUS activity, mice were orally administered fluorescein di‐β‐D‐glucuronide (FDGlcU, 7.3 µmol kg^−1^, 0.1 mL per mouse) (CAS: 129787‐66‐2, BioRuler, CT, United States) following the final XLP administration. Mice were then euthanized, and fluorescence imaging was conducted using an IVIS Spectrum imaging system (Bruker) with 470 nm excitation/535 nm emission filters, as previously described.^[^ ^32^ ^]^

To evaluate the inhibitory activity of XLP's main active components on GUS, 4‐MUG was used as a substrate probe, and the absorbance of its hydrolysis product, 4‐methylumbelliferone, was measured. The experiment was conducted using a pre‐cooled 96‐well plate to rapidly prepare the reaction system, which consisted of phosphate buffer (pH 6.8), GUS solution (40 µL, 500 ng mL^−1^), 4‐MUG solution (30 µL, 500 µM), and the test monomer solution. The final test monomer concentrations ranged from 0 to 225 µM. After mixing, the reaction system was immediately placed in a spectrophotometer (OD 405 nm), followed by incubation at 37 °C for 30 min, with a second absorbance measurement taken afterward. Residual GUS activity (%) was calculated as: Residual activity (%) = 100% – [OD (drug 30 min) – OD (drug 0 min)] / [OD (control 30 min) – OD (control 0 min)].

To measure fecal GUS activity, PNPG was used as a substrate probe. Frozen fecal pellets were homogenized in potassium phosphate buffer (pH 7.4, 0.01 M) and centrifuged (10 000 × g, 20 min). Protein concentration was determined using a BCA Protein Assay Kit (Yeasen Biotech, Shanghai, China). A 20 µL aliquot of protein (100 µg mL^−1^) from each fecal homogenate was incubated with 20 µL of PNPG (0.5 mM) and 80 µL potassium phosphate buffer for 30 min at 37 °C. The reaction was stopped by adding 250 µL of 0.5 M sodium hydroxide, and p‐nitrophenol levels were detected using a microplate reader at OD 405 nm.

To assess the GUS activity produced by *L. reuteri*, 4‐MUG was used as a substrate probe, and the inhibitory effect of XLP was measured based on the absorbance of its hydrolysis product. The reaction system (500 µL) contained phosphate buffer (pH 6.8), *L. reuteri* suspension (200 µL, OD600 = 0.1), 4‐MUG solution (150 µL, 500 µM), and XLP solution (100, 200, 400 µg mL^−1^). Absorbance was measured at 405 nm before and after incubation at 37 °C for 30 min. Additionally, images were captured under white light and UV light (366 nm) to visualize GUS enzyme activity.

### Detection of GUS‐Producing Bacteria in Feces

To detect GUS‐producing bacteria, a 4‐MUG agar culture plate was used following previously described methods.^[^ ^32^ ^]^ Fecal pellets (50–70 mg) were homogenized and centrifuged at 376 × g for 5 min at room temperature. The supernatant was then subjected to a second centrifugation at 9391 × g for 20 min, and the resulting precipitate was resuspended in 0.95% saline (500 µL). This bacterial suspension was then spread onto nutrient agar plates containing 4‐MUG and incubated at 37 °C for 12 h. The presence of 4‐methylumbelliferone, produced by GUS‐expressing bacteria, was detected under UV light (366 nm).

### Partial Least Squares Path Modeling (PLS‐PM) Analysis

An optimized PLS‐PM algorithm was developed by integrating methodologies from Qi X et al.^[^ ^38^ ^]^ with modifications to better quantify the effects of XLP intervention in CPT11‐induced intestinal mucositis. The model assessed four key parameters: (1) Inflammatory response, evaluated through colon length measurements and cytokine levels; (2) Epithelial regeneration, represented by goblet cell markers and stem cell markers; (3) GUS module, indicated by GUS [EC: 3.2.1.31] expression and GUS enzymatic activity; (4) *L. reuteri* abundance, determined via RT‐qPCR analysis of murine fecal samples.

### Molecular Docking Simulation

The 3D structure of the GUS protein (PDB: 3K46) was retrieved from the RCSB PDB database. AutoDock Tools v1.5.6 was used for initial protein structure processing, including removal of water molecules, addition of hydrogen atoms, and conversion to a pdbqt file for docking analysis. The 2D structures of the GUS inhibitor (PubChem SID: 482 821 795), berberine (PubChem CID: 12 456), jateorhizine (PubChem CID: 72 323), coptisine (PubChem CID: 72 322), groenlandicine (PubChem CID: 3 084 708), magnoflorine (PubChem CID: 73 337), costunolide (PubChem CID: 5 281 437), and palmatine (PubChem CID: 19 009) were obtained from PubChem and converted into Mol2 files using OpenBabel software. Hydrogen atoms were added, charges were assigned, and torsional flexibility was defined for each compound to optimize molecular docking. Docking calculations were conducted using AutoDock Tools, with the grid box centered at (‐14.604, ‐32.129, 47.104) and dimensions set to 80 × 60 × 60. Following docking, post‐docking optimizations were performed using PyMOL v1.8 Molecular Graphics System.

### Drug Synergy Index

The synergistic effect efficiency of SN38 with XLP and its active components was evaluated using the combination index (CI), calculated via the probability sum method. The formula used was: E'_a+b_ = E_a_ + E_b_ – (E_a_ * E_b_), where Ea and Eb represent the individual effects of each drug. Additionally, the synergy index (Q) was determined using the formula: Q = [E_a+b_] / [E'_a+b_]. The interpretation of Q values is provided in Table S4 (Supporting Information), while the significance of Q value ranges is detailed in Table S5 (Supporting Information).

### Statistical Analyses

Data analysis was performed using GraphPad Prism 9.0 (GraphPad Software, La Jolla, CA, USA), with results expressed as mean ± standard deviation (SD) or standard error of the mean (SEM). Statistical comparisons between groups were conducted using unpaired Student's t‐test or one‐way analysis of variance (ANOVA). At least three independent experiments were performed. Spearman correlation analysis was used to assess correlations, and differences with P < 0.05 were considered statistically significant (*p < 0.05; **p < 0.01; **p < 0.001; *ns, no significance*). Clinical data cited in this study were obtained from the European Nucleotide Archive (https://www.ebi.ac.uk/ena/browser/home, Project: PRJNA494824). 16S rDNA sequencing data were analyzed on Majorbio Cloud (www.majorbio.com/). Heatmaps of transcriptomic analysis of colon tissue were generated using Heatmapper (www.heatmapper.ca/expression/).

## Conflict of Interest

The authors declare no conflict of interest.

## Author Contributions

B.Y., R.G., and L.Z. contributed equally to this work. B.Y., R.G., and L.Z. conceived and designed the experiments. B.Y., R.G., D.L., C.L., Z.W., F.A., B.Z., Z.Y., X.G., H.W., and K.W. performed the material preparation, data collection, and analysis. B.Y., W.D., and R.G. wrote the first draft of the manuscript. K.C., C.L., Z.W., and W.D. supervised the study and revised the manuscript.

## Supporting information



Supporting Information

## Data Availability

The data are available from the corresponding author on reasonable request. Microbiological data available at NCBI via BioProject ID PRJNA797942.
