# Supplementary material for: β‐Glucuronidase‐Expressing Lactobacillus reuteri Triggers Irinotecan Enterotoxicity Through Depleting the Regenerative Epithelial Stem/Progenitor Pool
Source: Adv Sci (Weinh). 2025 Apr 26;12(26):2411052. doi: 10.1002/advs.202411052 (PMC12245021; doi:10.1002/advs.202411052)
Supplement: Supplementary file 1 — Supporting Information [file ADVS-12-2411052-s001.docx]

**Supporting Information**

**β-glucuronidase-Expressing *Lactobacillus reuteri* Triggers Irinotecan Enterotoxicity through Depleting the Regenerative Epithelial Stem/Progenitor Pool**

Bei Yue, Ruiyang Gao, Ling Zhao, Donghui Liu, Cheng Lv, Ziyi Wang, Fangbin Ai, Beibei Zhang, Zhilun Yu, Xiaolong Geng, Hao Wang, Kang Wang, Kaixian Chen, Chenghai Liu *, Zhengtao Wang *, and Wei Dou *

**
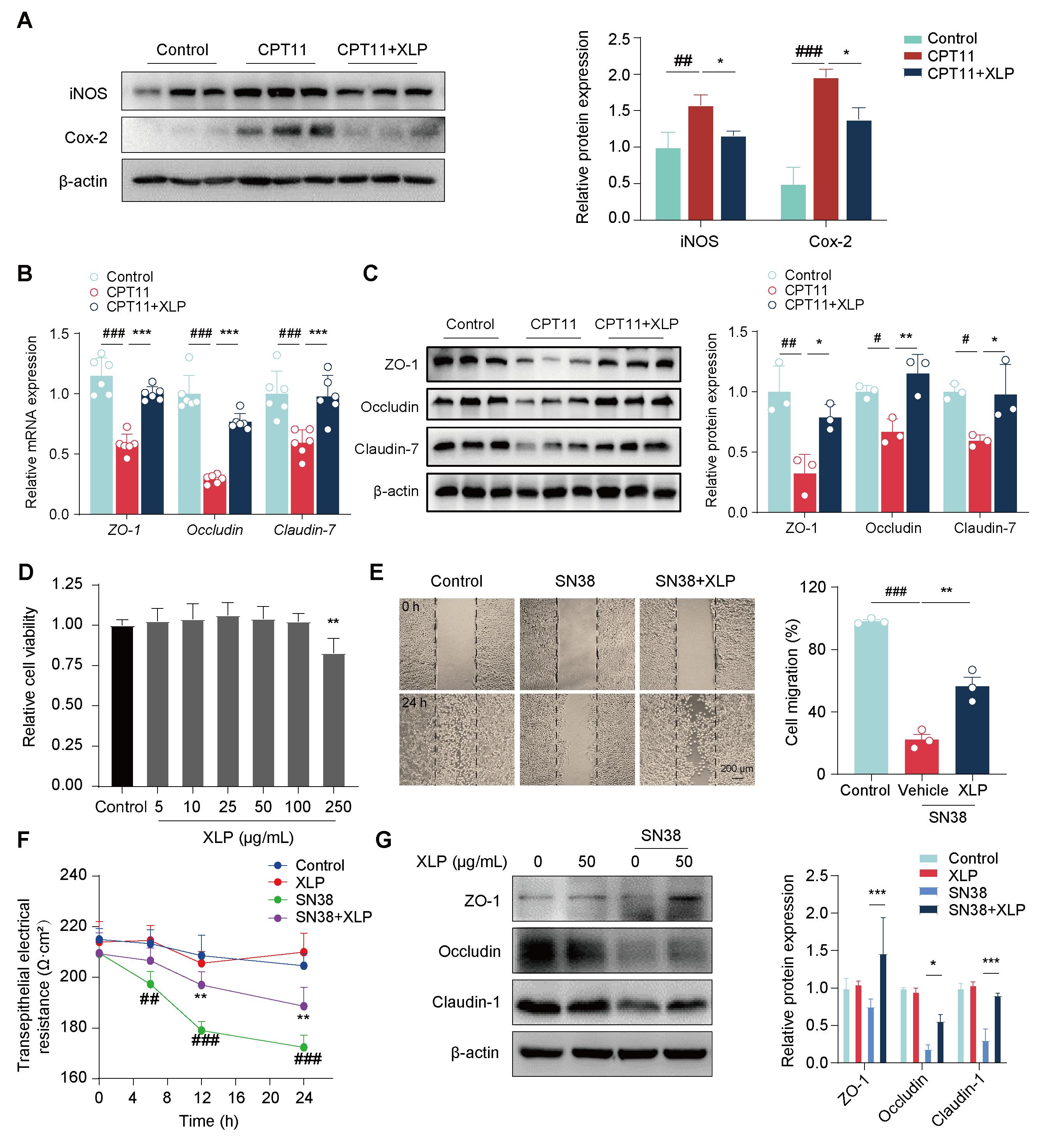
**

**Fig. S1.** XLP inhibited pro-inflammatory mediators in CPT11-induced enteritis mice. A) Representative western blots and quantitative analysis of iNOS and Cox-2 in colon tissues. B) Relative mRNA levels of ZO-1, Occludin, and Claudin-7 in colon tissues across each group (n = 6). C) Representative western blots and quantitative analysis of ZO-1, Occludin, and Claudin-7 in colon tissues (n = 3). D) Effects of XLP on NCM460 cell viability. Cells were treated with XLP (0, 5, 10, 25, 50, 100, 250 μg/mL) for 24 h, and cell viability was measured (n = 6). E) Representative images of the scratch wound healing assay in NCM460 cells treated with SN38 (500 nM) alone or SN38 (500 nM) + XLP (50 μg/mL) at 0 and 24 h post-treatment (n = 3). F) NCM460 cell monolayers were treated with XLP (50 μg/mL), SN38 (500 nM), or SN38 (500 nM) + XLP (50 μg/mL) for 0, 6, 12 and 24 h, respectively; the TEER were then measured (n = 3). G) Representative western blots and quantitative analysis of ZO-1, Occludin, and Claudin-1 in colon tissues. Data are expressed as mean ± SD (n = 3). Statistical analysis was performed using one-way ANOVA. ^##^*P* < 0.01, ^###^*P* < 0.001 vs. Control group; **P* < 0.05, ***P* < 0.01, ****P* < 0.001 vs. CPT11/SN38 group.

**
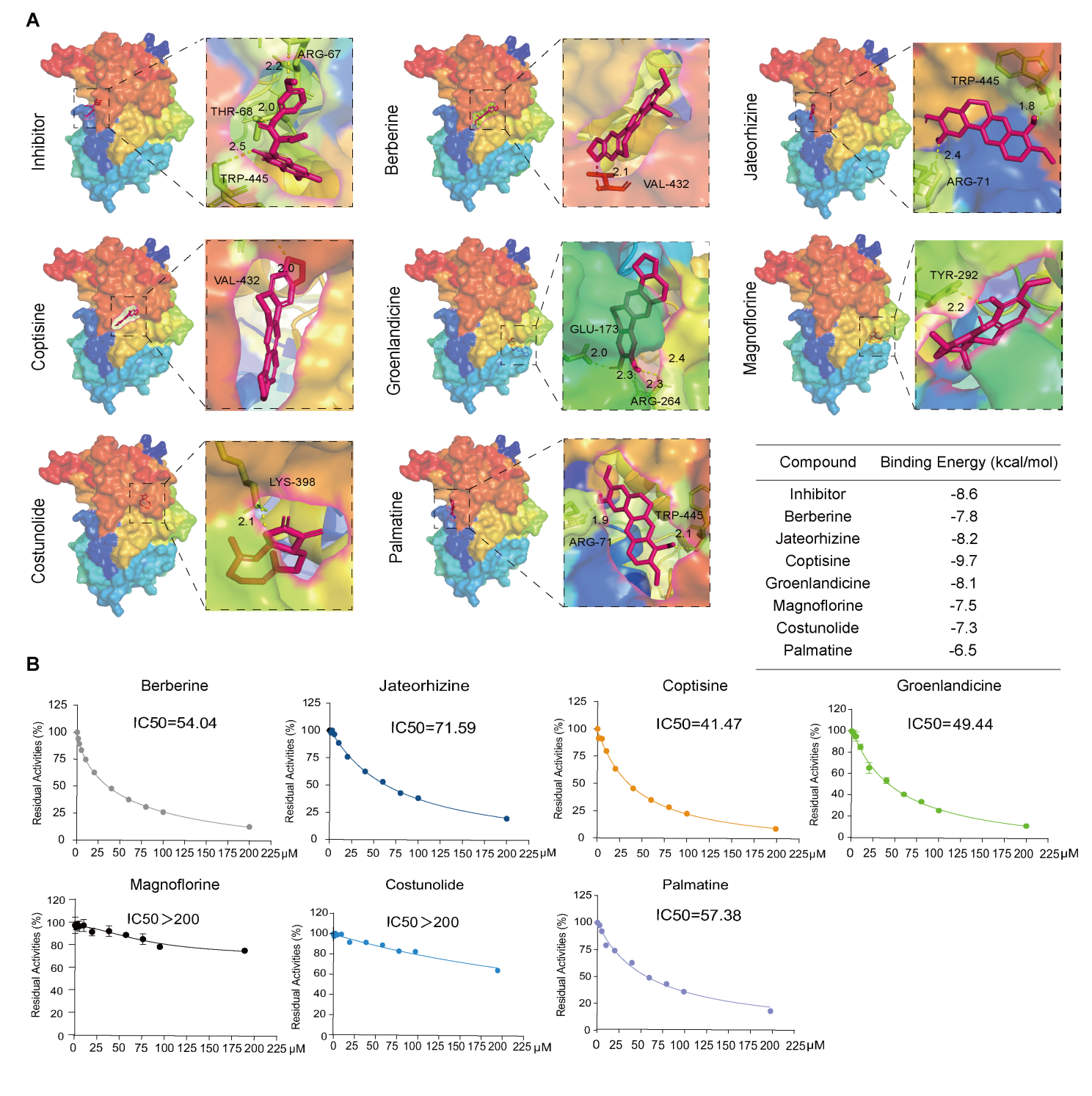
**

**Fig. S2.** XLP suppressed the activity of GUS enzyme. A) A stereo view of the binding model of *E. coli* GUS (PDB: 3K46) combined with inhibitors, including berberine, jateorhizine, coptisine, groenlandicine, magnoflorine, costunolide, and palmatine, along with their respective binding energies. B) The inhibitory effects of berberine, jateorhizine, coptisine, groenlandicine, magnoflorine, costunolide, and palmatine on *E. coli* GUS enzyme were determined by the 4-MUG method (n = 3). Data are expressed as mean ± SD.

**
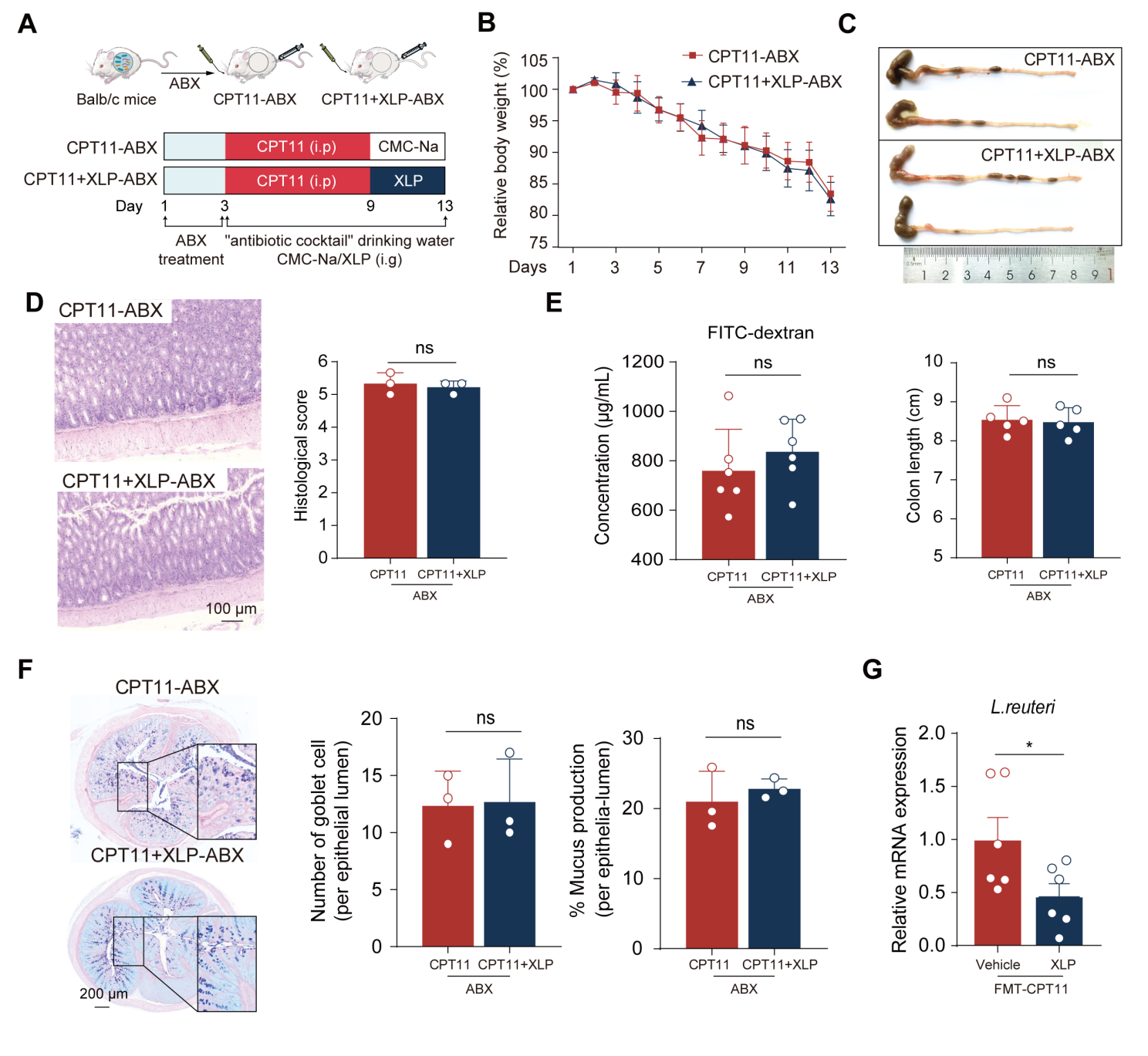
**

**Fig. S3.** Antibiotics treatment abolished the beneficial effect of XLP on CPT11-induced mice enteritis. A) Schematic diagram of the experimental procedure. B) Body weight was recorded following the animal experiment. Data were plotted as a percentage of basal body weight. Data are expressed as mean ± SEM (n = 6). C) The representative colon was photographed, and colon length was measured (n = 5). D) Representative colon H&E staining and histological score analysis (scale bar = 100 μm, n = 3). E) Serum concentrations of FITC-dextran in mice (n = 6). F) Representative AB-PAS staining of the colonic sections (scale bar = 200 μm) and the counting of goblet cells per villus calculation unit, and the mucus production of per epithelia-lumen (n = 3). G) The mRNA expression level of *L. reuteri* in feces was detected by RT-qPCR. Data are expressed as mean ± SD (n = 6). Statistical analysis was performed using an unpaired Student's *t*-test. **P* < 0.05 *vs.* FMT-CPT11 group. ns, not significant.

**
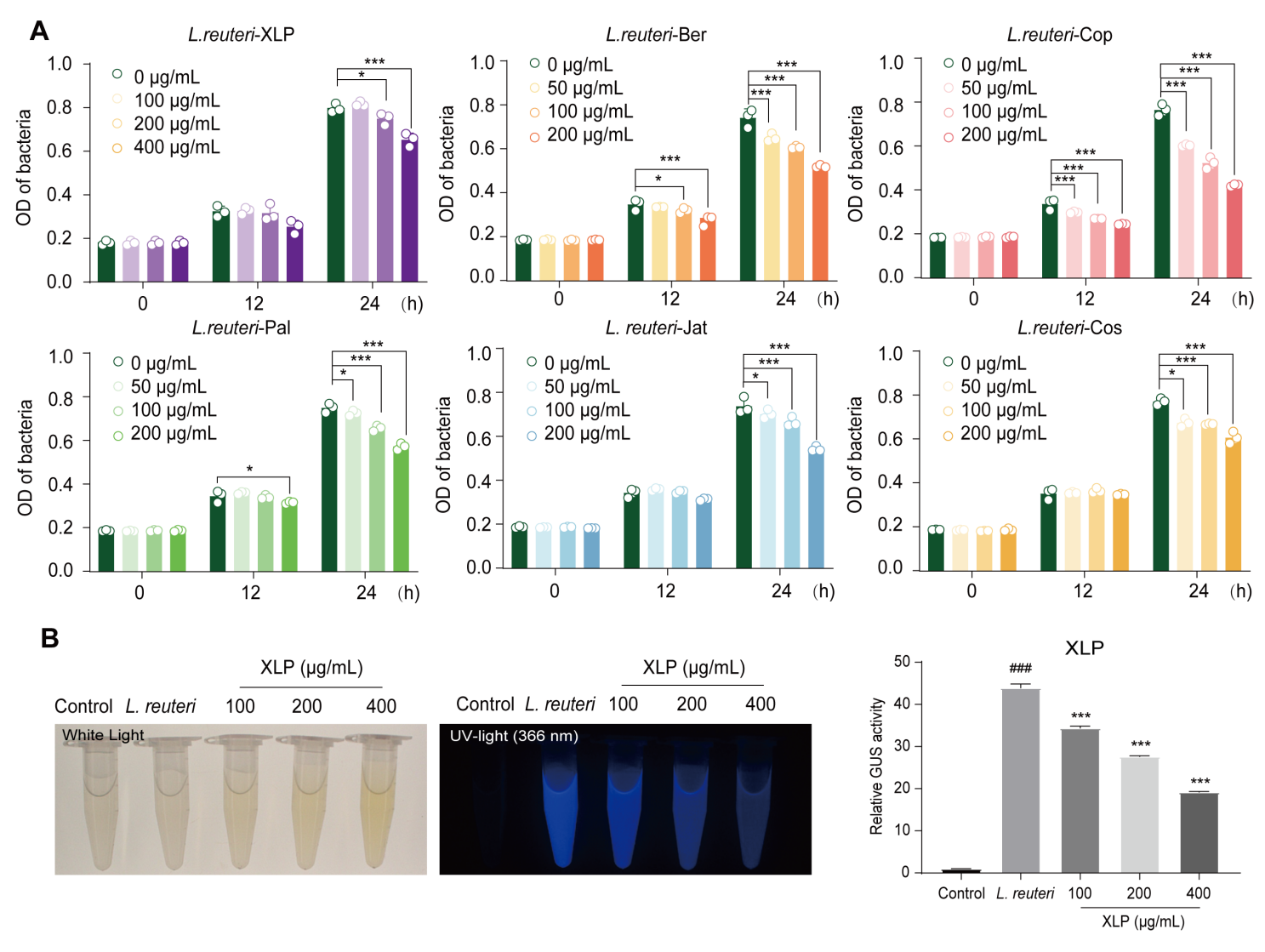
**

**Fig. S4.** XLP and its major active components suppressed the propagation of *L. reuteri in vitro.* A) Effects of XLP and its major active components (berberine (Ber), coptisine (Cop), palmatine (Pal), jateorhizine (Jat), and costunolide (Cos)) on the propagation of *L. reuteri* by *in vitro* co-incubation experiment (n = 3). B) The impact of XLP on GUS-producing bacteria *L. reuteri.* The intensity of the blue coloration corresponds to the abundance of *L. reuteri* bacteria, with brighter shades indicating higher abundance (n = 3). Data are expressed as mean ± SD. Statistical analysis was performed using one-way ANOVA. **P* < 0.05, ****P* < 0.001 vs. Control group (A). ^###^*P* < 0.001 vs. Control group; ****P* < 0.001 vs. *L. reuteri* group (B).

**
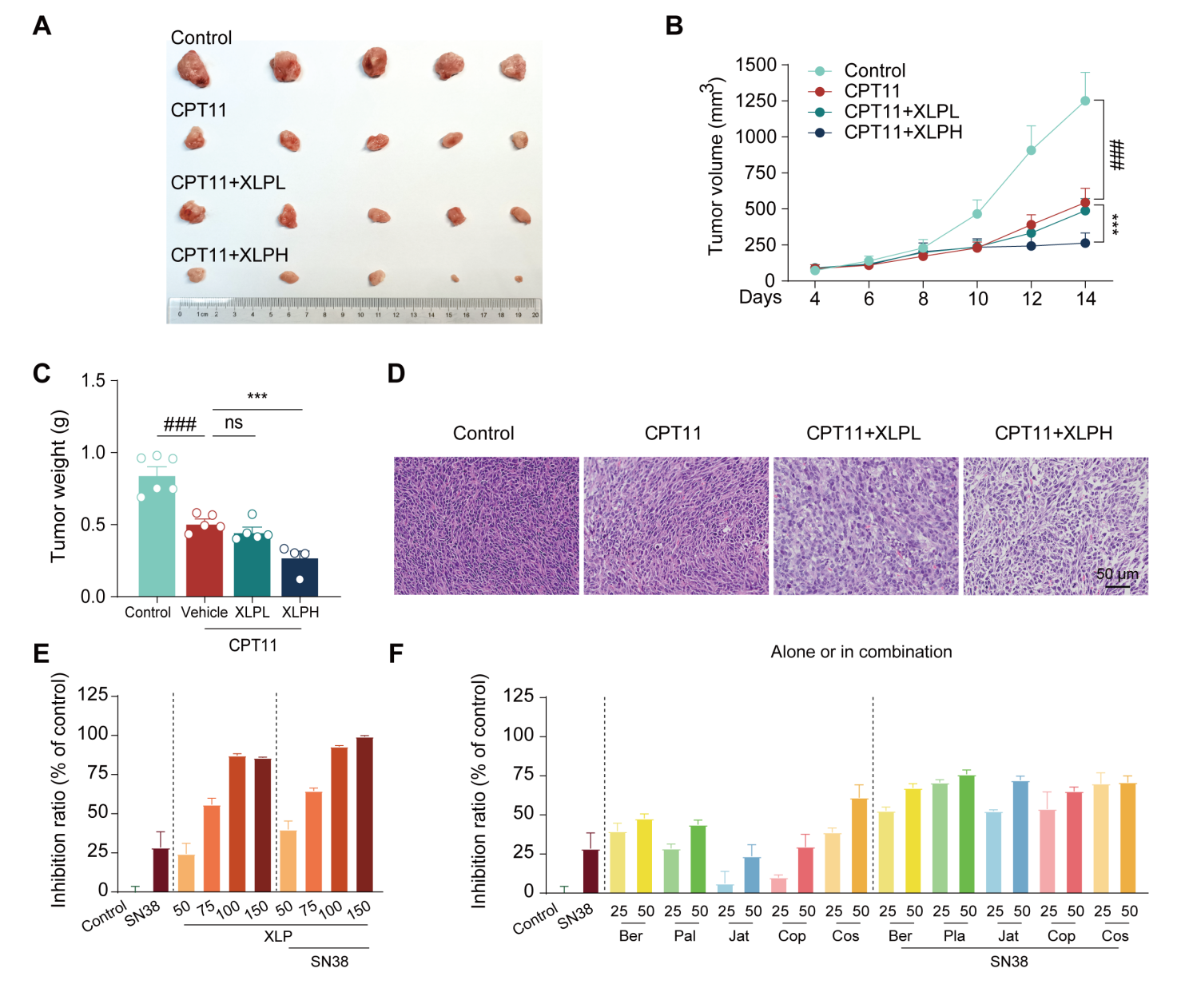
**

**Fig. S5.** XLP synergistically enhanced the anticancer efficacy of CPT11 in a CT26 colon cancer xenograft model. A) Representative gross images of the CRC tumor (n = 5). B) The volume of CRC tumors in mice during a 2-week administration of CPT11, CPT11+XLPL, or CPT11+XLPH (n = 6). C) The weight of CRC tumors in mice with the intervention of CPT11, CPT11+XLPL, or CPT11+XLPH (n = 5-6). D) Representative images of tumor tissue stained by H&E (scale bar = 50 μm). E) The cytotoxic effects of SN38 (500 nM), XLP (50, 75, 100, and 150 mg/mL), or SN38 combined with XLP on CT26 cells (n = 3). F) The cytotoxic effects of SN38 (500 nM), the main active compounds of XLP (berberine, palmatine, jateorhizine, coptisine, and costunolide of 25 and 50 μM), or SN38 combined with the main active compounds of XLP (berberine, palmatine, jateorhizine, coptisine, and costunolide) on CT26 cells (n = 3). Data are expressed as mean ± SD. Statistical analysis was performed using one-way ANOVA. *^##^P* < 0.01 *vs*. Control group, *^###^P* < 0.001 *vs*. Control group; **P* < 0.05, ***P* < 0.01, ****P* < 0 .001 *vs*. CPT11 group.

**Table S1.** Taxa composition of dominant bacteria (abundance ≥ 0.01%) at the family level and major GUS-producing bacteria at the genus level.

| Taxonomy | Control | CPT11 | CPT11+XLP | *p*-value | *p*-value |
| --- | --- | --- | --- | --- | --- |
|  | (Mean±SEM) | (Mean±SEM) | (Mean±SEM) | (CPT11 vs. Con) | (CPT11+XLP vs. CPT11) |
| *Bacteroidaceae* | 0.041±0.012 | 0.084±0.047 | 0.387±0.093 | 0.5815 | <0.0001 |
| *Muribaculaceae* | 0.265±0.067 | 0.089±0.023 | 0.094±0.016 | 0.0008 | 0.9935 |
| *Lactobacillaceae* | 0.167±0.121 | 0.197±0.091 | 0.074±0.021 | 0.7727 | 0.0233 |
| *Lachnospiraceae* | 0.175±0.050 | 0.084±0.019 | 0.116±0.047 | 0.1134 | 0.7376 |
| *Akkermansiaceae* | 4.450e-005±4.450e-005 | 0.062±0.025 | 0.221±0.080 | 0.3421 | 0.0027 |
| *Prevotellaceae* | 0.036±0.011 | 0.136±0.044 | 0.003±0.003 | 0.0769 | 0.013 |
| *Rikenellaceae* | 0.073±0.023 | 0.026±0.011 | 0.007±0.003 | 0.5325 | 0.8969 |
| *Helicobacteraceae* | 0.015±0.004 | 0.031±0.007 | 0.025±0.015 | 0.9289 | 0.9911 |
| *Oscillospiraceae* | 0.034±0.012 | 0.014±0.004 | 0.012±0.003 | 0.8937 | 0.9983 |
| *Desulfovibrionaceae* | 0.009±0.002 | 0.015±0.003 | 0.014±0.005 | 0.9889 | 0.9999 |
| *Ruminococcaceae* | 0.011±0.004 | 0.016±0.005 | 0.009±0.003 | 0.9924 | 0.9841 |
| *Bacillaceae* | 0.017±0.017 | 0.022±0.012 | 0.000±0.000 | 0.9944 | 0.8712 |
| *o_Clostridia_UCG-014* | 0.017±0.005 | 0.013±0.008 | 3.553e-004±2.376e-004 | 0.996 | 0.9514 |
| *Micrococcaceae* | 0.010±0.010 | 0.012±0.007 | 2.283e-005±2.283e-005 | 0.9989 | 0.96 |
| *Clostridiaceae* | 4.996e-004±2.771e-004 | 0.012±0.007 | 3.530e-004±2.238e-004 | 0.9631 | 0.9622 |
| *g_Lactobacillus* | 4.769±2.648 | 13.711±4.799 | 6.356±2.206 | < 0.0001 | 0.0001 |
| *g_Escherichia-Shigella* | 0.042±0.040 | 0.204±0.157 | 4.833e-004±4.833e-004 | 0.9914 | 0.9866 |
| *g_Streptococcus* | 0.058±0.015 | 0.132±0.011 | 0.025±0.006 | 0.9985 | 0.9963 |
| *g_Cutibacterium* | 0.000±0.000 | 0.002±0.001 | 0.000±0.000 | >0.9999 | >0.9999 |
| *g_Corynebacterium* | 0.000±0.000 | 0.010±0.002 | 0.000±0.000 | >0.9999 | >0.9999 |
| *g_Roseburia* | 1.775±0.494 | 0.392±0.099 | 1.773±0.767 | 0.5952 | 0.6325 |

Note: The color gradient indicates microbial abundance, with orange representing high abundance and blue representing low

abundance. Additionally, significant differences in p-values are denoted by red font.

**Table S2.** The inhibitory effects of SN38, berberine (Ber), palmatine (Pal), jateorhizine (Jat), coptisine (Cop), and costunolide (Cos) alone, or SN38 in combination with XLP and its active compounds, on CT26 mouse colorectal cancer cells, along with the calculation of the combination index.

| Treatment | Inhibition rate (%) | Combined CI (Q) | Combined effectiveness |
| --- | --- | --- | --- |
| SN38 500 nM | 28.49 | — | — |
| Ber 25 μM | 39.60 | — | — |
| Ber 50 μM | 47.71 | — | — |
| Pal 25 μM | 28.54 | — | — |
| Pal 50 μM | 43.82 | — | — |
| Jat 25 μM | 6.25 | — | — |
| Jat 50 μM | 23.63 | — | — |
| Cop 25 μM | 10.15 | — | — |
| Cop 50 μM | 29.74 | — | — |
| Cos 25 μM | 38.94 | — | — |
| Cos 50 μM | 61.19 | — | — |
| XLP 50 μg/mL | 24.45 | — | — |
| XLP 75 μg/mL | 55.88 | — | — |
| XLP 100 μg/mL | 87.26 | — | — |
| XLP 150 μg/mL | 85.79 | — | — |
| SN38 500 nM+Ber 25 μM | 52.60 | 0.93 | additive effect |
| SN38 500 nM+Ber 50 μM | 67.33 | 1.08 | additive effect |
| SN38 500 nM+Pal 25 μM | 70.83 | 1.45 | enhanced |
| SN38 500 nM+Pal 50 μM | 75.95 | 1.27 | enhanced |
| SN38 500 nM+Jat 25 μM | 52.41 | 1.59 | enhanced |
| SN38 500 nM+Jat 50 μM | 72.24 | 1.59 | enhanced |
| SN38 500 nM+Cop 25 μM | 53.91 | 1.51 | enhanced |
| SN38 500 nM+Cop 50 μM | 65.26 | 1.31 | enhanced |
| SN38 500 nM+Cos 25 μM | 70.16 | 0.97 | additive effect |
| SN38 500 nM+Cos 50 μM | 70.98 | 1.26 | enhanced |
| SN38 500 nM+XLP 50 μg/mL | 39.90 | 1.16 | enhanced |
| SN38 500 nM+XLP 75 μg/mL | 64.59 | 1.88 | enhanced |
| SN38 500 nM+XLP 100 μg/mL | 92.96 | 2.70 | enhanced |
| SN38 500 nM+XLP 150 μg/mL | 99.32 | 2.89 | enhanced |

**Table S3.** The list of primers used for RT-qPCR.

| Gene | Forward primer (5’-3’) | Reverse primer (5’-3’) |
| --- | --- | --- |
| *m Cox-2* | GCCTTCCCTACTTCACAA | ACAACTCTTTTCTCATTTCCAC |
| *m Il-6* | GGCGGATCGGATGTTGTGAT | GGACCCCAGACAATCGGTTG |
| *m Tnf-α* | CTCTTCTCATTCCTGCTTGT | GTGGTTTGTGAGTGTGAGG |
| *m ZO-1* | GCCGCTAAGAGCACAGCAA | TCCCCACTCTGAAAATGAGGA |
| *m Claudin-7* | GGCCACTCGAGCCTTAATGGTG | CCTGCCCAGCCGATAAAGATGG |
| *m Occludin* | ATGTCCGGCCGATGCTCTC | TTTGCTGCTCTTGGGTCTGTAT |
| *m Muc2* | TGCCCACCTCCTCAAAGAC | TAGTTTCCGTTGGAACAGTGAA |
| *m Muc3a* | TGAGCAAAGGCAGTATCGTG | GCCTCCTTCTTGCATGTCTC |
| *m Muc5ac* | CCTCTCAGAGGAATGTGACTCTGCGC | CCAGGCAGCCACACTTCTCAACCT |
| *m Muc6* | TGCATGCTCAATGGTATGGT | TGTGGGCTCTGGAGAAGAGT |
| *m Muc13* | TCTGGACTCTGGCCACTCTT | GAGGACAGAGCCAGTCCAAG |
| *m Klf4* | AGGAACTCTCTCACATGAAGCG | GGTCGTTGAACTCCTCGGTC |
| *m Tff3* | CAGATTACGTTGGCCTGTCTCC | ATGCTTGCTACCCTTGGACCAC |
| *m Lgr5* | CCTACTCGAAGACTTACCCAGT | GCATTGGGGTGAATGATAGCA |
| *m Bmi1* | CCCCACTTAATGTGTGTCCTG | TTGCTGGTCTCCAAGTAACG |
| *m Ascl2* | AGGACGCAATAAGCTAAGCATC | AGTGGACGTTTGCACCTTCACG |
| *m β-defensin1* | GGCTGCCACCACTATGAAAACTC | GAGACAGAATCCTCCATGTTGAA |
| *m β-defensin2* | CTCCACCTGCAGCTTTTAGC | GCTAGGGAGCACTTGTTTGC |
| *m Lysozyme (Lyz)1* | GAGACCGAAGCACCGACTATG | CGGTTTTGACATTGTGTTCGC |
| *m Dclk1* | TGAACAAGAAGACGGCTCACTCC | GCTGGTGGGTGATGGACTTGG |
| *m Trpm5* | CCTCCGTGCTTTTTGAACTCC | CATAGCCAAAGGTCGTTCCTC |
| *m β-actin* | CTGTGCCCATCTACGAGGGCTAT | TTTGATGTCACGCACGATTTCC |
| *g_Lactobacillus* | TGGAAACAGRTGCTAATACCG | GTCCATTGTGGAAGATTCCC |
| *L. reuteri* | CAGACAATCTTTGATTGTTTAG | GCTTGTTGGTTTGGGCTCTTC |
| *L.casei* | TTTGAGGGGACGACCCTCAAGCA | CGCCGACAAGCTATGAATTCACTTG |
| *L. paracasei* | GGATTGGGTTTTGCGTGATGGTCGC | TGCATTTCCCCGCTTTCATGACT |
| *L. acidophilus* | TGCAAAGTGGTAGCGTAAGC | CCTTTCCCTCACGGTACTG |
| *L. fermentum* | ACTAACTTGACTGATCTACGA | TTCACTGCTCAAGTAATCATC |
| *L. gasseri* | AATACTCCCGAAGCACGTCA | TCATTGTGTTTGGCAATCGT |
| *All bacteria* | CGGTGAATACGTTCCCGG | TACGGCTACCTTGTTACGACTT |

**Table S4.** Interpretation of the formula indices.

| Index | Index meaning |
| --- | --- |
| E_a_ | Drug efficacy achieved when drug a is applied alone |
| E_b_ | Drug efficacy achieved when b drug is applied alone |
| E’_a+b_ | The drug efficacy achieved by the combination of drug a and drug b is the expected effect of the combination |
| E_a+b_ | The actual efficacy of drug a combined with drug b |
| Q | The synergistic index of two synergistic drugs |

**Table S5.** Significance of Q value ranges.

| Q range | Significance of Q value ranges |
| --- | --- |
| <0.55 | Drug a and b have obvious antagonism (1 + 1 << 2) |
| 0.55-0.85 | Drug a is antagonistic to drug b (1 + 1 < 2) |
| 0.85-1.15 | The effects of drug a and b can be simply added together (1 + 1 = 2) |
| 1.15-20 | The combined efficacy of drug a and drug b was enhanced (1 + 1> 2) |
| >20 | The combined efficacy of drug a and drug b was significantly enhanced (1 + 1 >> 2) |
